# Supplementary material for: Rock glaciers across the United States predominantly accelerate coincident with rise in air temperatures
Source: Nat Commun. 2024 Aug 31;15:7581. doi: 10.1038/s41467-024-52093-z (PMC11365947; doi:10.1038/s41467-024-52093-z)
Supplement: Supplementary file 1 — Supplementary Information [file 41467_2024_52093_MOESM1_ESM.pdf]

Supplementary Information to

Rock glaciers across the United States predominantly accelerate coincident with rise in air temperatures

Andreas Kääb, Julie Røste
Department of Geosciences, University of Oslo, Norway; kaeaeb@geo.uio.no

This Supplementary Information contains: page

- Air temperatures at SNOTEL stations close to rock glaciers ……………….. 2
- Snow cover data ……………………………………………………………... 3
- Air temperatures and precipitation from the PRISM data set ……………….. 5
- Comparison of speed time series with meteorological data …………………. 11
- Speed time series …………………………………………………………….. 17
- Information sheets for all rock glaciers ……………………………………… 18
- List of airphotos ……………………………………………………………… 32

Air temperatures at SNOTEL stations close to rock glaciers

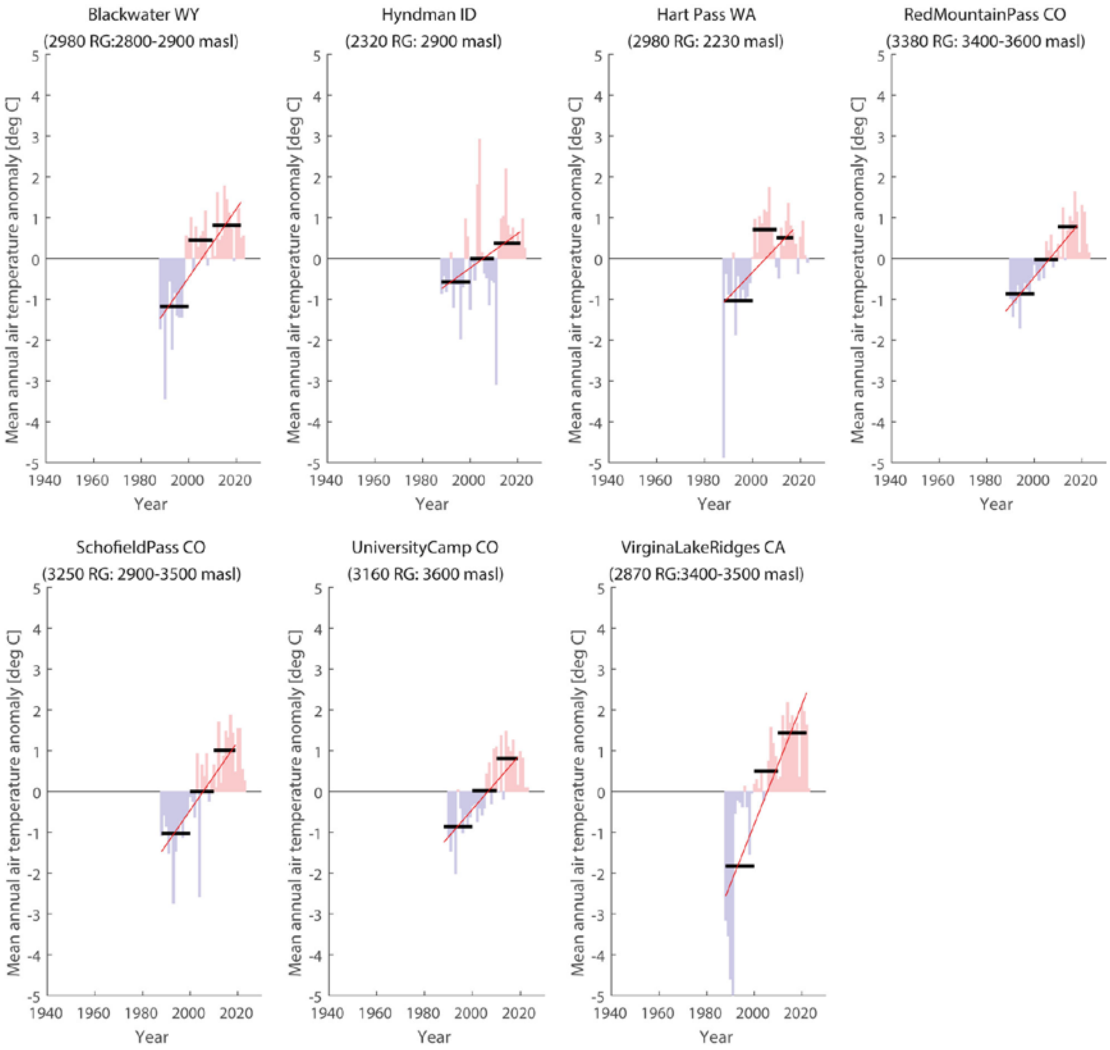

Figure S1: Annual mean air temperature anomalies at Snow Telemetry Network (SNOTEL) stations against average of entire measurement period (1988–2023), decadal means (black lines), and trend lines (red lines). The numbers below the station name are the station elevation, and the elevation range of the closest rock glacier (RG). Approximate horizontal distances between SNOTEL stations and rock glaciers: Blackwater (station) – Galena/Sulphur/Crater (rock glaciers): 30-40 km, Hyndman – Old Hyndman: 7 km, Harts Pass – Star Peak: 50 km, Red Mountain Pass – Sneffels/Teakettle/Mears/Twin Sisters: 15 km, Schofield Pass – Ferguson/Thomas: 30 km, South Lake (Fig S2) – Pine/Birch/Cardinal: 15-22 km, Virginia Lake Ridges – Pine/Birch/Cardinal: 120 km, University Camp – Arapaho: 5 km. SNOTEL data are obtained from the National Water and Climate Center (NWCC) by the Natural Resources Conservation Service (NRCS), U.S. Department of Agriculture. WA:Washington, CA: California, ID: Idaho, WY: Wyoming, CO: Colorado.

Snow cover data

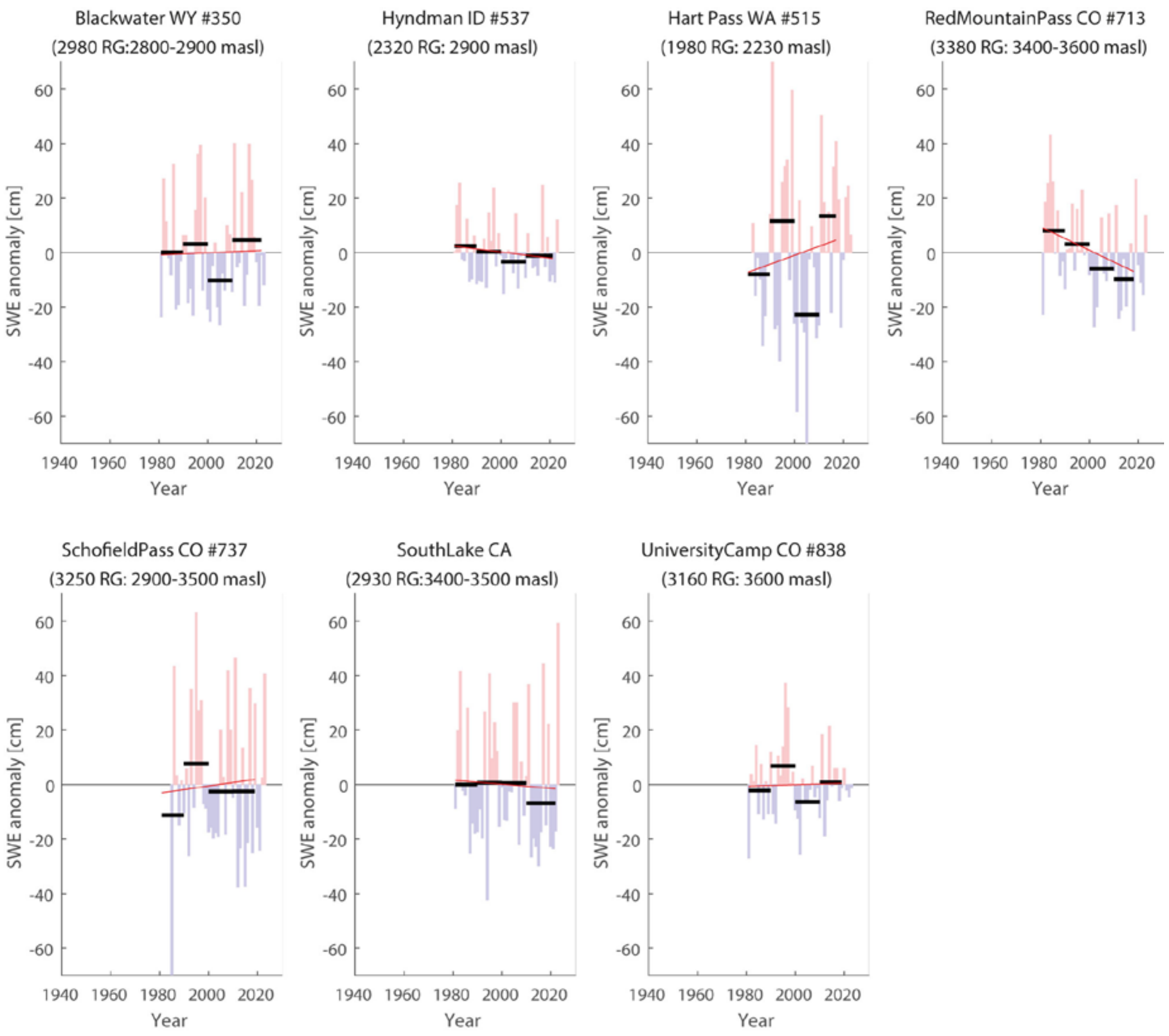

Figure S2: Maximum monthly snow water equivalent (SWE) per water year at SNOTEL stations against average of entire measurement period (1981–2023), decadal means (black lines), and trend lines (red lines). The numbers below the station name are the station elevation, and the elevation (range) of the closest rock glacier (RG). For horizontal distances to rock glaciers and data source see caption of Fig. S1.

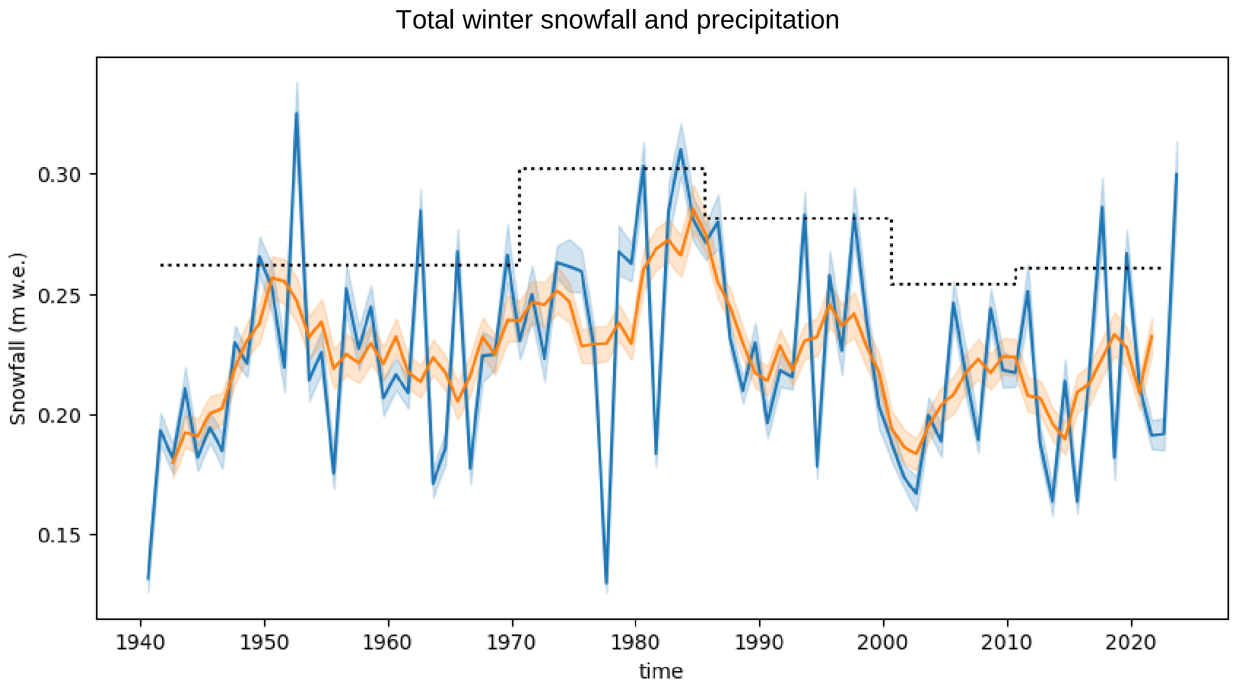

Figure S3: European Centre for Medium-Range Weather Forecasts Reanalysis (ERA) 5 total winter (Oct-Mar) snowfall above 2000m for the Western U.S. (blue) with error band from all cells above 2000m. 5-year rolling mean (orange) with error band from all cells above 2000m. Binned temporal average is of winter precipitation (not only snowfall; dotted black). ERA5 data from Hersbach et al. (2023) (see reference list of main article).

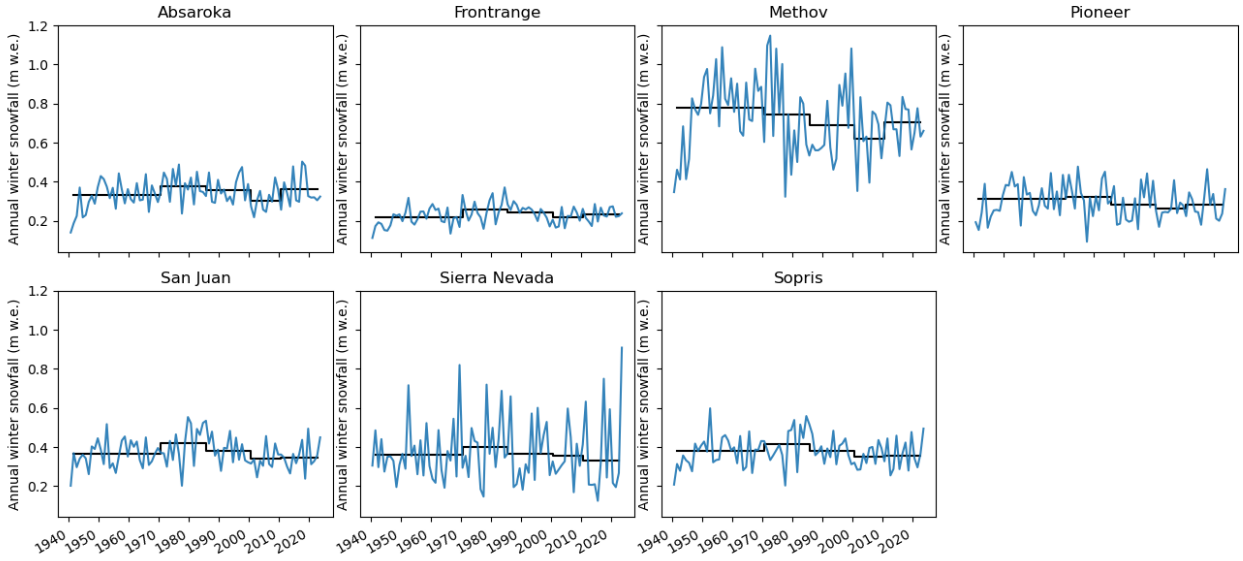

Figure S4: Total winter snowfall for closest ERA5-cell to given mountain range location (blue). Binned temporal average of winter snowfall (black). ERA5 data from Hersbach et al. (2023) (see reference list of main article).

Air temperatures and precipitation from the PRISM data set

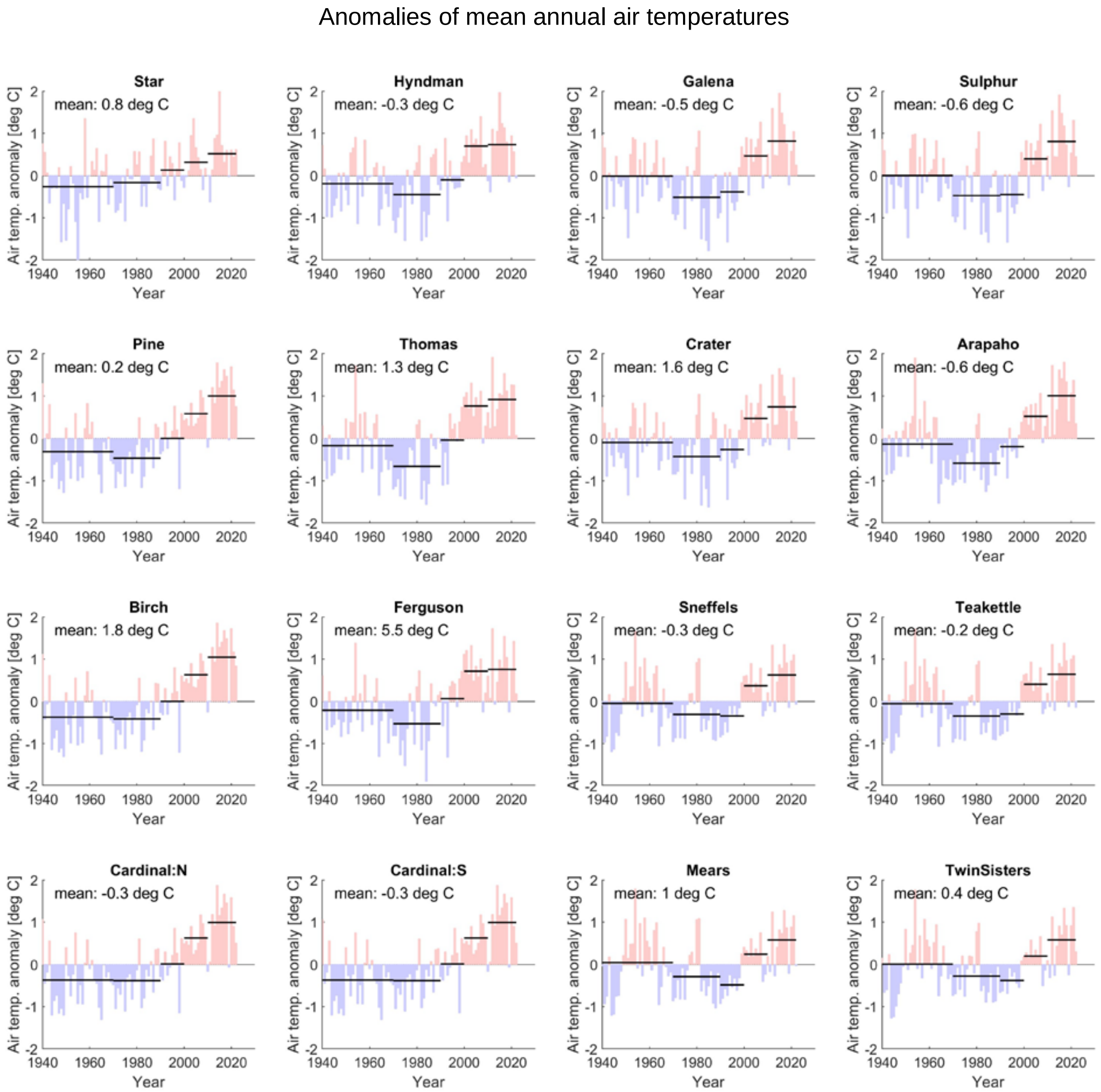

Figure S5: Anomalies of mean annual air temperatures and their decadal-scale means (black lines; 1940-1970, 1970-1990, 1990-2000, 2000-2010, 2010-2022) interpolated at rock glacier locations from the Parameter-elevation Regressions on Independent Slopes Model (PRISM) data set (PRISM Climate Group, Oregon State University, https://prism.oregonstate.edu, accessed 6 Jun 2024). Mean temperatures are means of PRISM monthly means. N: North, S: South.

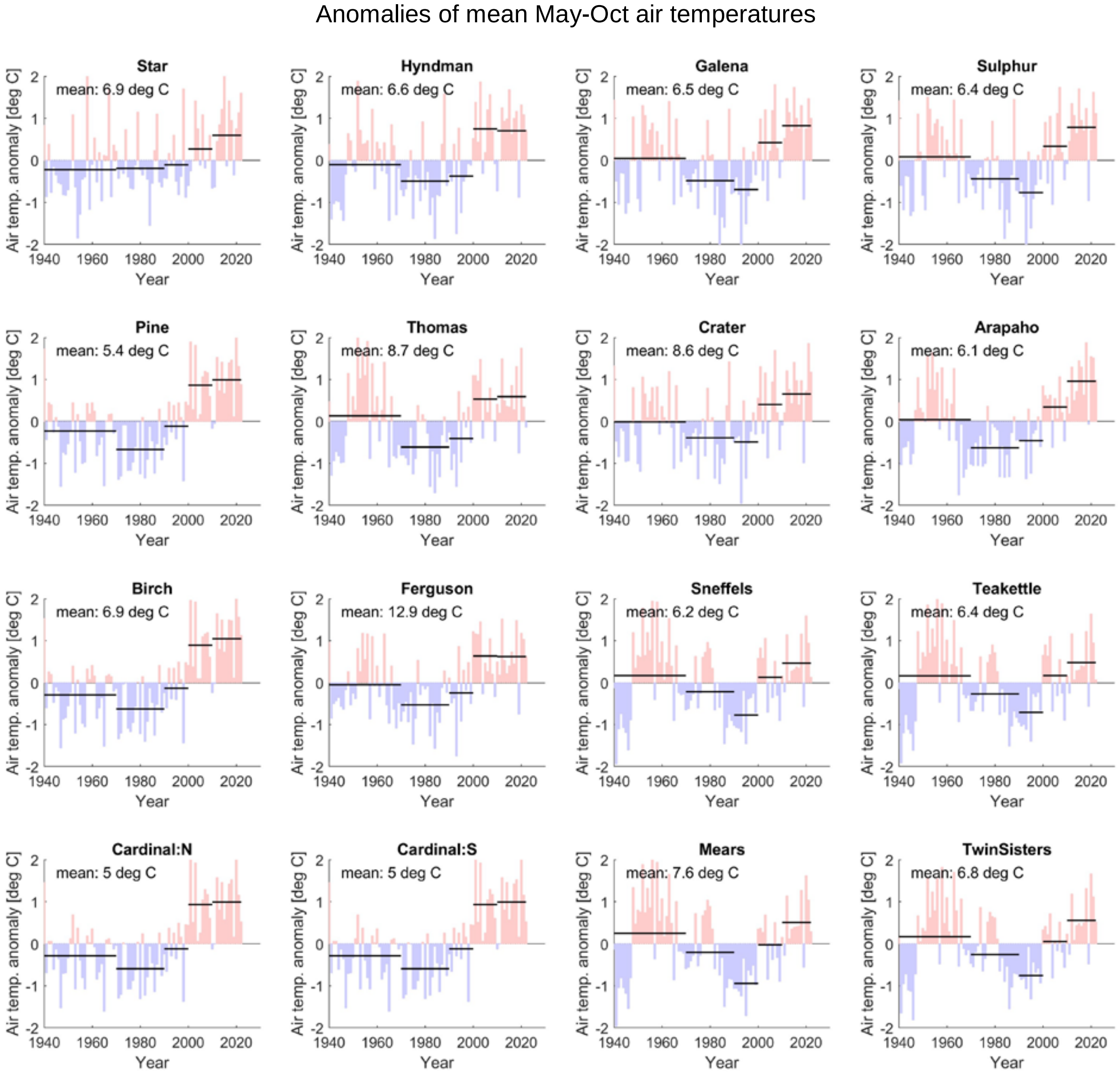

Figure S6: Anomalies of mean May-Oct air temperatures and their decadal means (black lines; 1940-1970, 1970-1990, 1990-2000, 2000-2010, 2010-2022) interpolated at rock glacier locations from the PRISM data set (PRISM Climate Group, Oregon State University, https://prism.oregonstate.edu). Mean temperatures are means of PRISM monthly means.

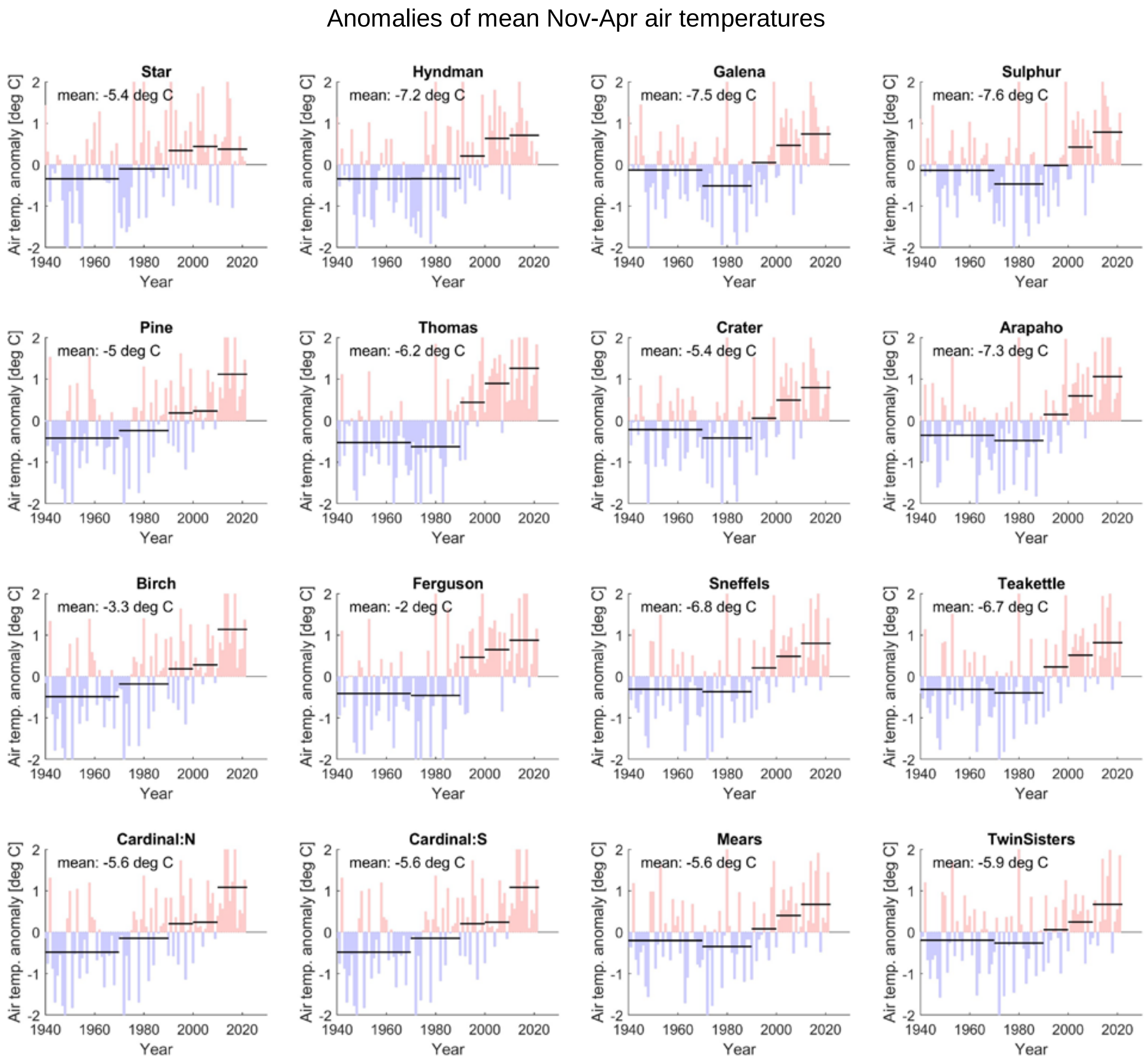

Figure S7: Anomalies of mean Nov-Apr air temperatures and their decadal means (black lines; 1940-1970, 1970-1990, 1990-2000, 2000-2010, 2010-2022) interpolated at rock glacier locations from the PRISM data set (PRISM Climate Group, Oregon State University, https://prism.oregonstate.edu). Mean temperatures are means of PRISM monthly means.

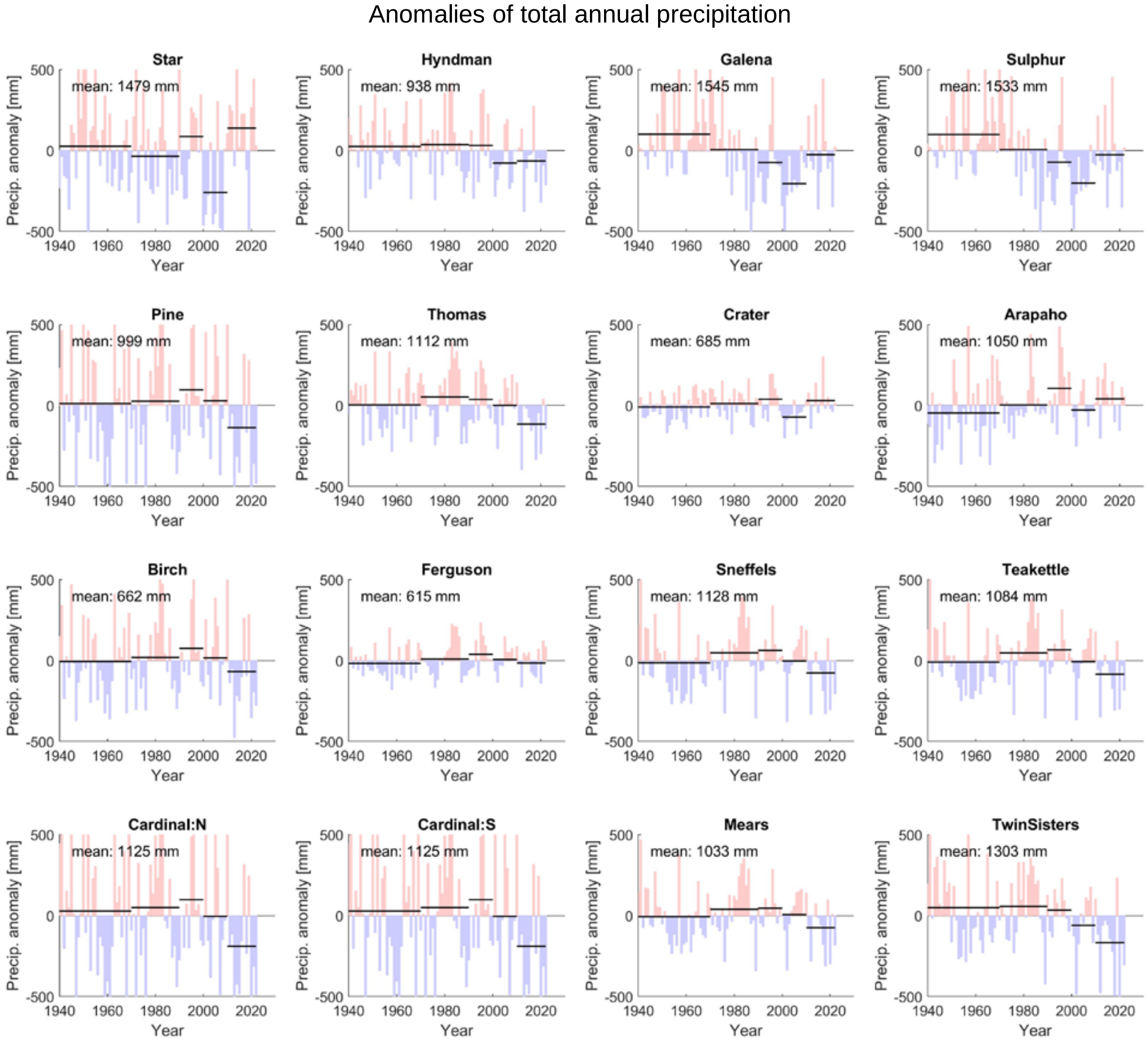

Figure S8: Anomalies of total annual precipitation and their decadal means (black lines; 1940-1970, 1970-1990, 1990-2000, 2000-2010, 2010-2022) interpolated at rock glacier locations from the PRISM data set (PRISM Climate Group, Oregon State University, https://prism.oregonstate.edu). Total precipitations are the sum of PRISM monthly means.

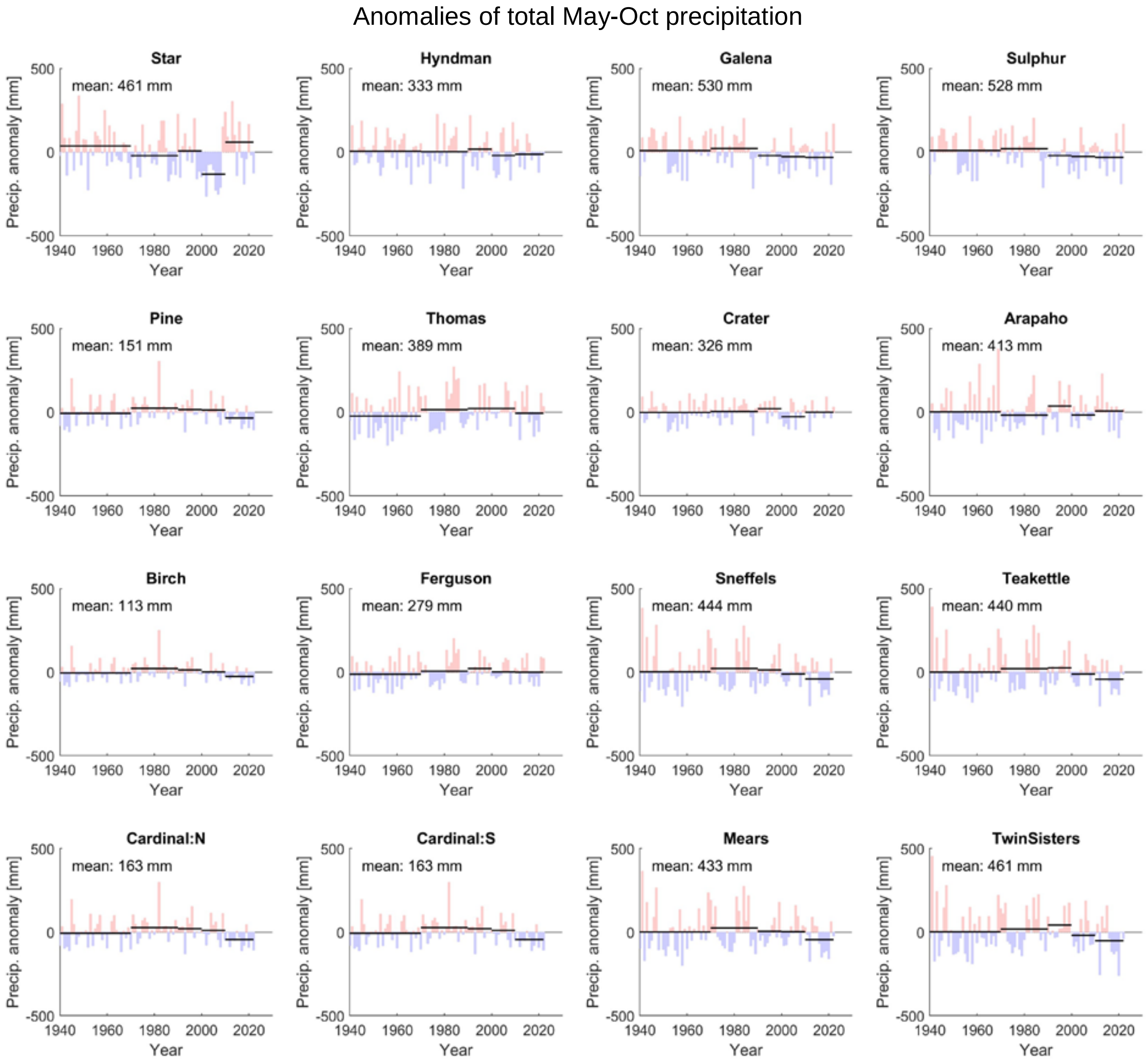

Figure S9: Anomalies of total May-Oct precipitation and their decadal means (black lines; 1940-1970, 1970-1990, 1990-2000, 2000-2010, 2010-2022) interpolated at rock glacier locations from the PRISM data set (PRISM Climate Group, Oregon State University, https://prism.oregonstate.edu). Total precipitations are the sum of PRISM monthly means.

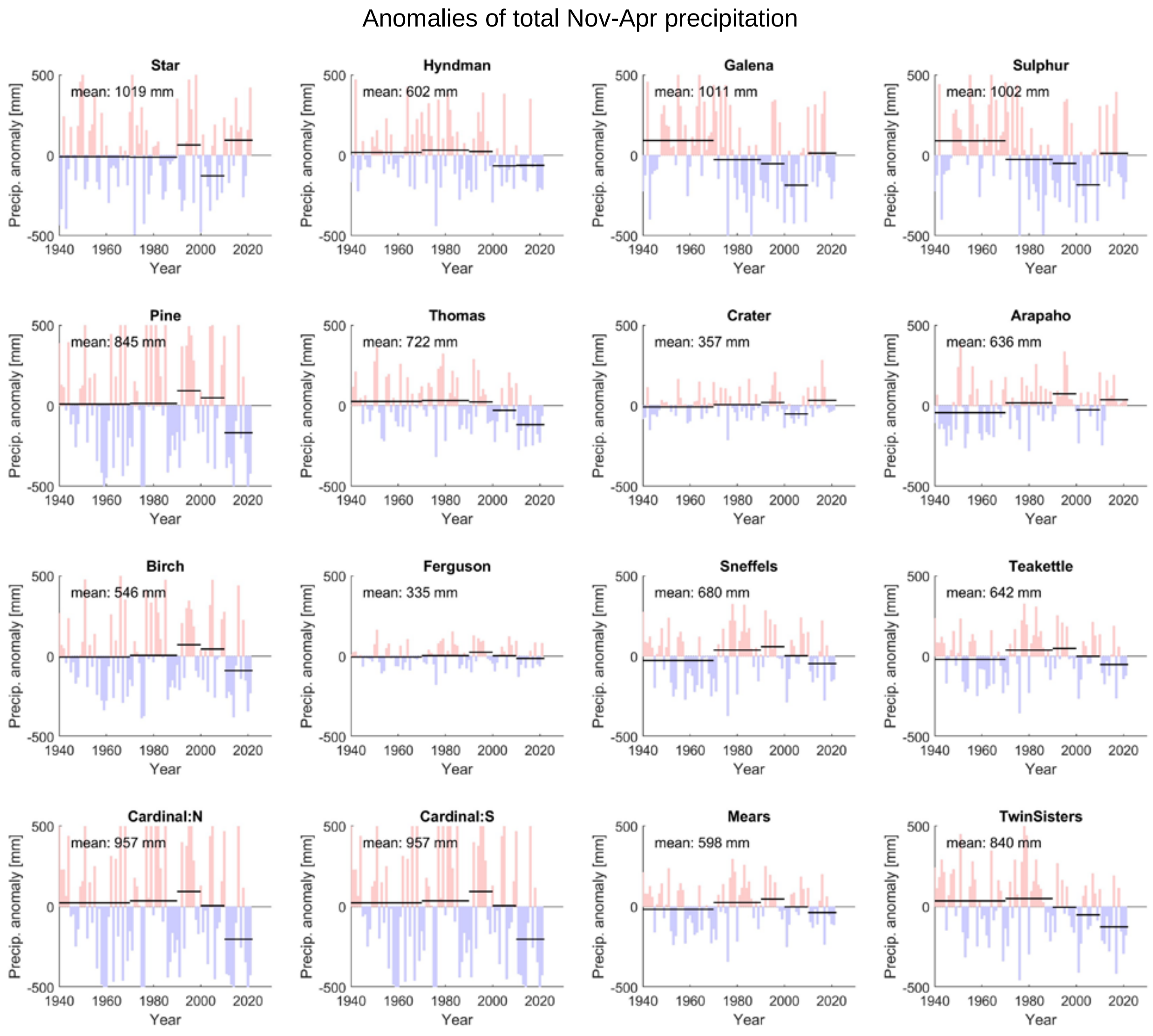

Figure S10: Anomalies of total Nov-Apr precipitation and their decadal means (black lines; 1940-1970, 1970-1990, 1990-2000, 2000-2010, 2010-2022) interpolated at rock glacier locations from the PRISM data set (PRISM Climate Group, Oregon State University, https://prism.oregonstate.edu). Total precipitations are the sum of PRISM monthly means.

Comparison between rock glaciers speeds and meteorological data

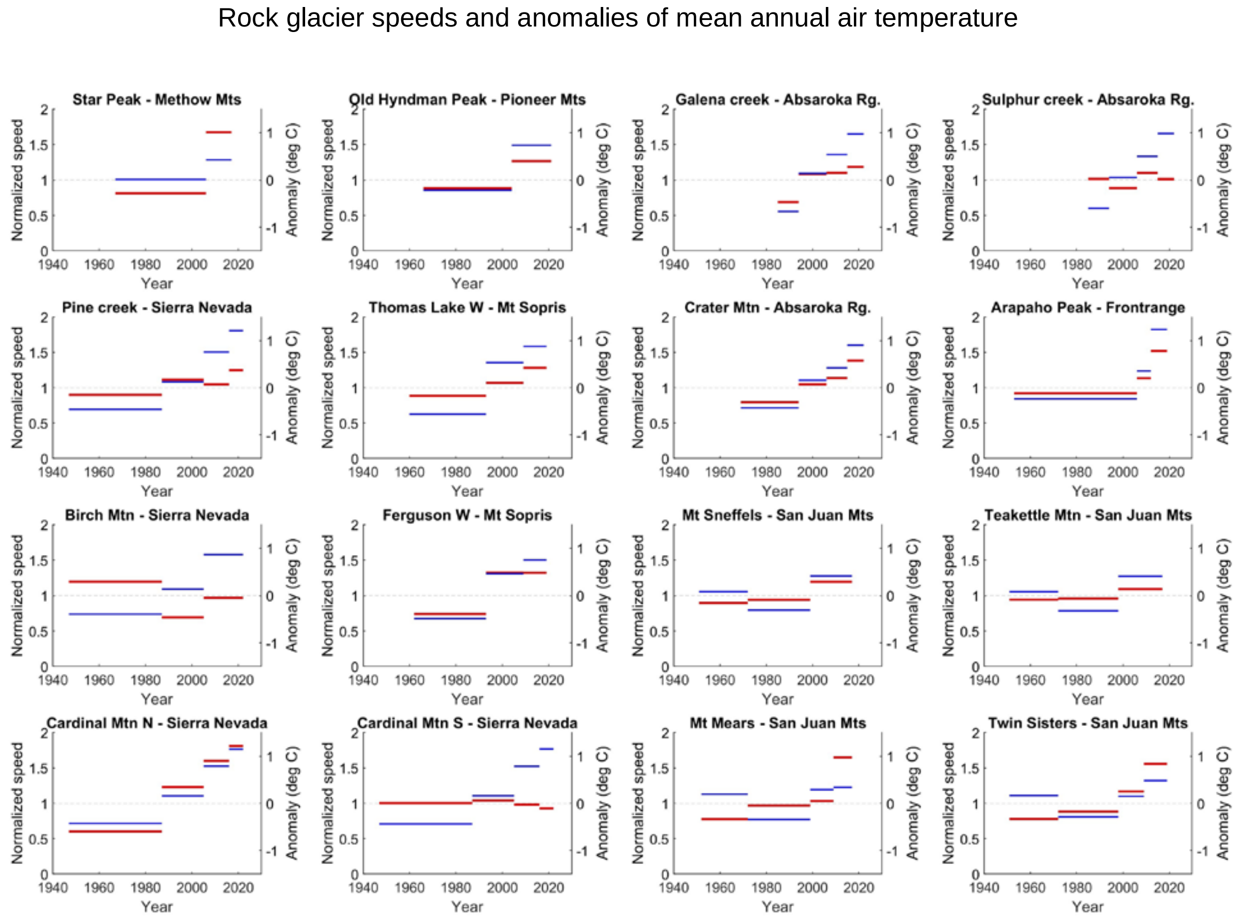

Figure S11: Rock glacier speed changes (red) and anomalies of mean annual air temperatures for the intervals of the speed measurements (blue). Rock glacier speeds are the same as in Fig.2, the air temperature anomaly means are computed for different intervals than in Fig. S5.

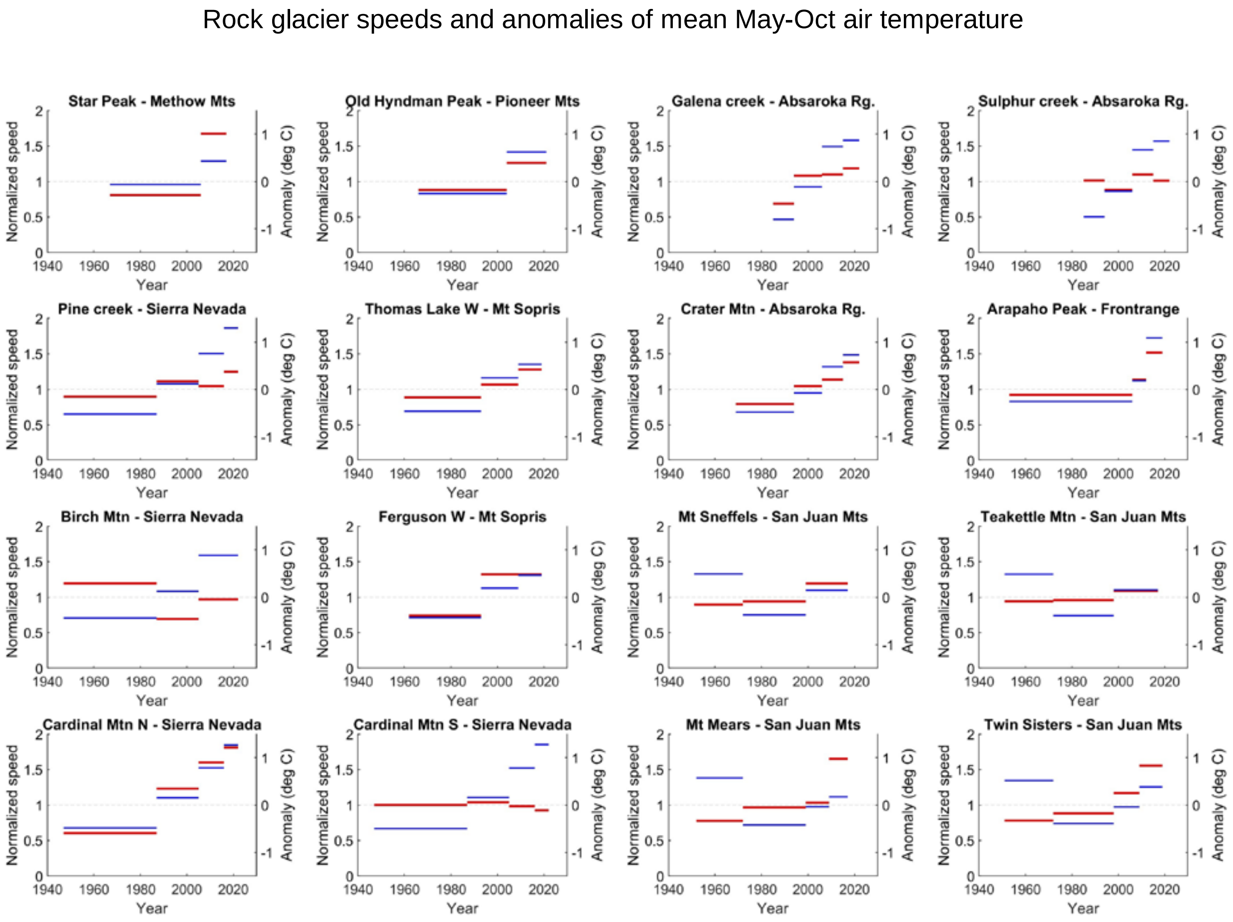

Figure S12: Rock glacier speed changes (red) and anomalies of mean May-Oct air temperatures for the intervals of the speed measurements (blue). Rock glacier speeds are the same as in Fig.2, air temperature anomaly means computed for different intervals than in Fig. S6.

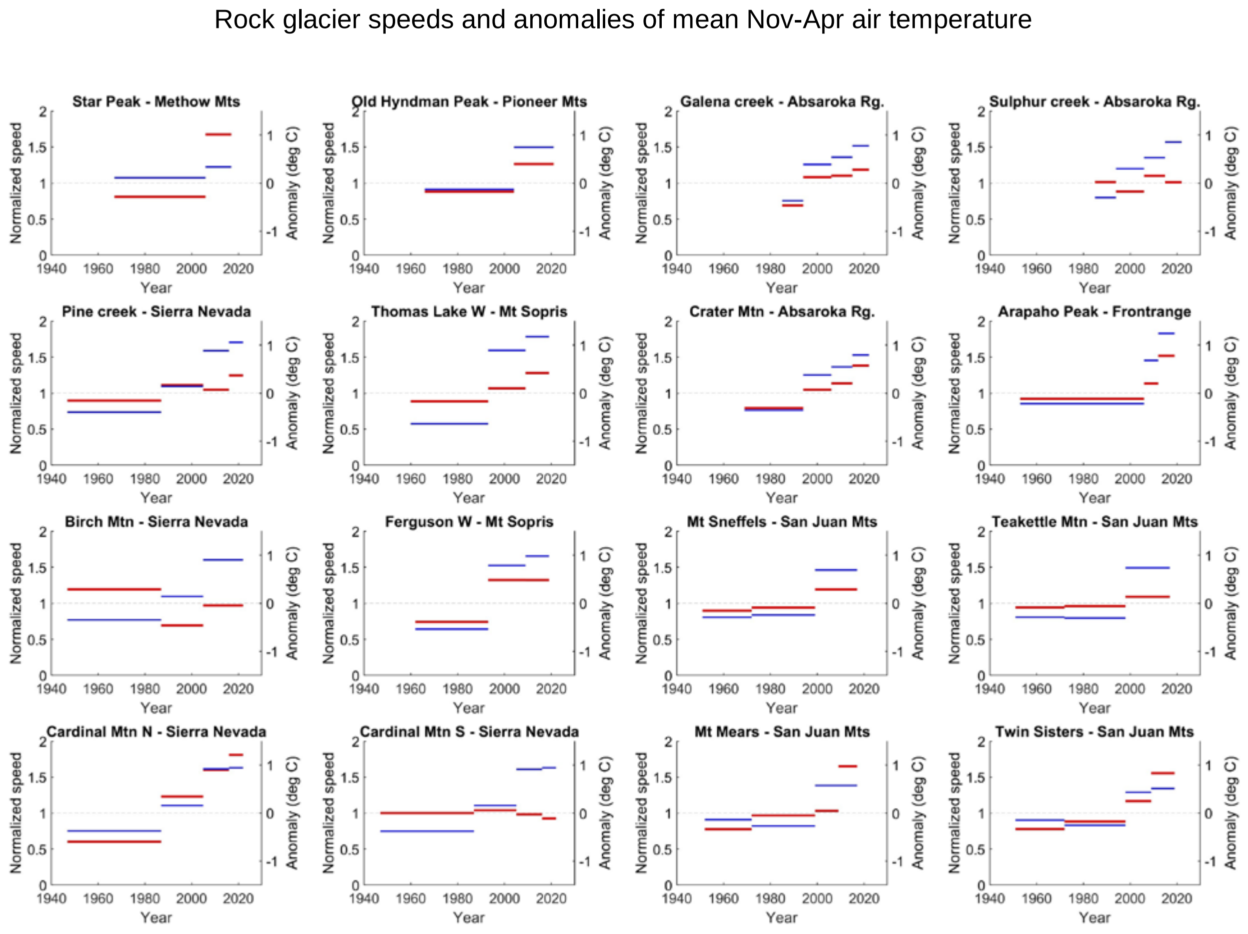

Figure S13: Rock glacier speed changes (red) and anomalies of mean Nov-Apr air temperatures for the intervals of the speed measurements (blue). Rock glacier speeds are the same as in Fig.2, air temperature anomaly means computed for different intervals than in Fig. S7.

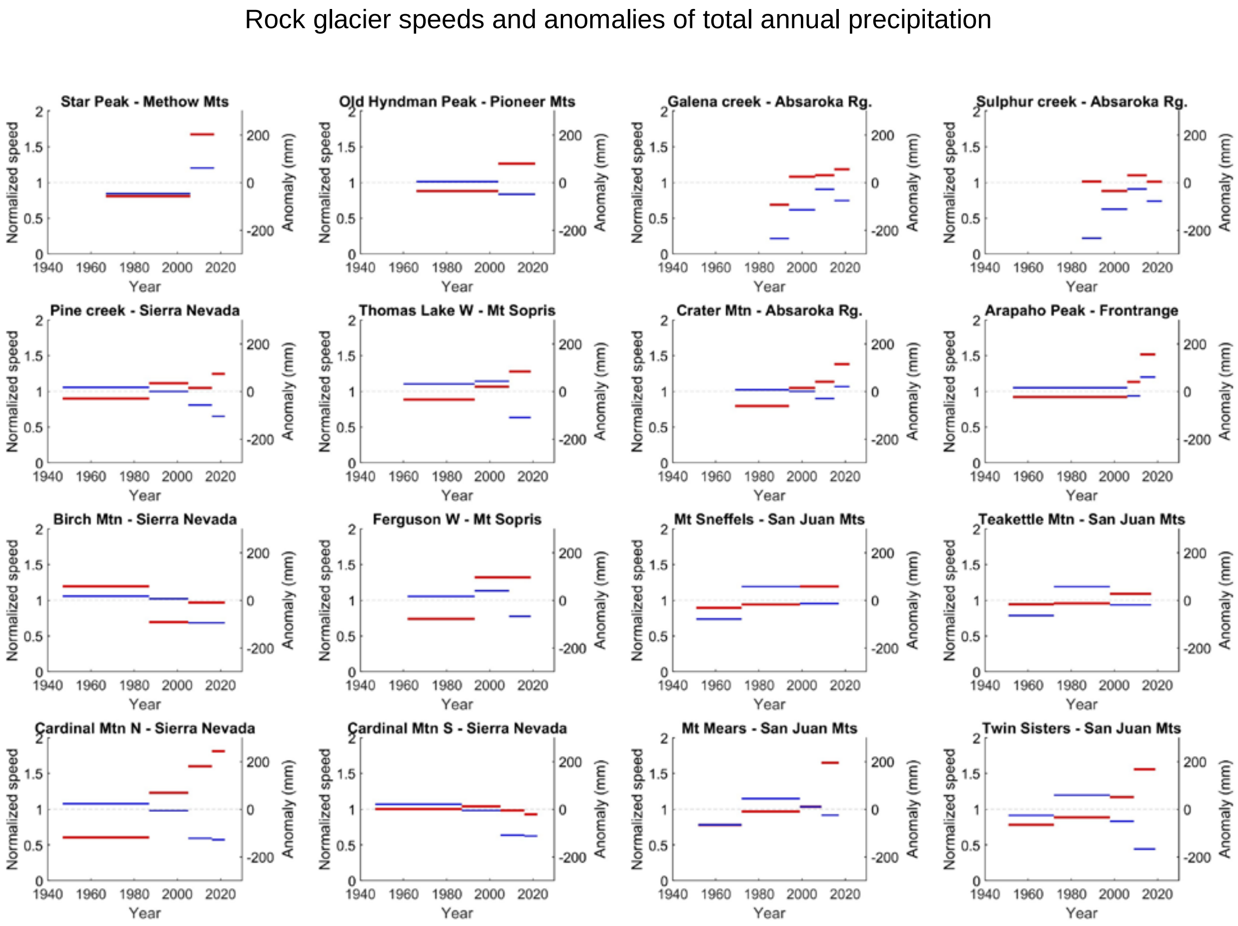

Figure S14: Rock glacier speed changes (red) and anomalies of total annual precipitation for the intervals of the speed measurements (blue). Rock glacier speeds are the same as in Fig.2, air temperature anomaly means computed for different intervals than in Fig. S8.

Rock glacier speeds and anomalies of total May-Oct precipitation

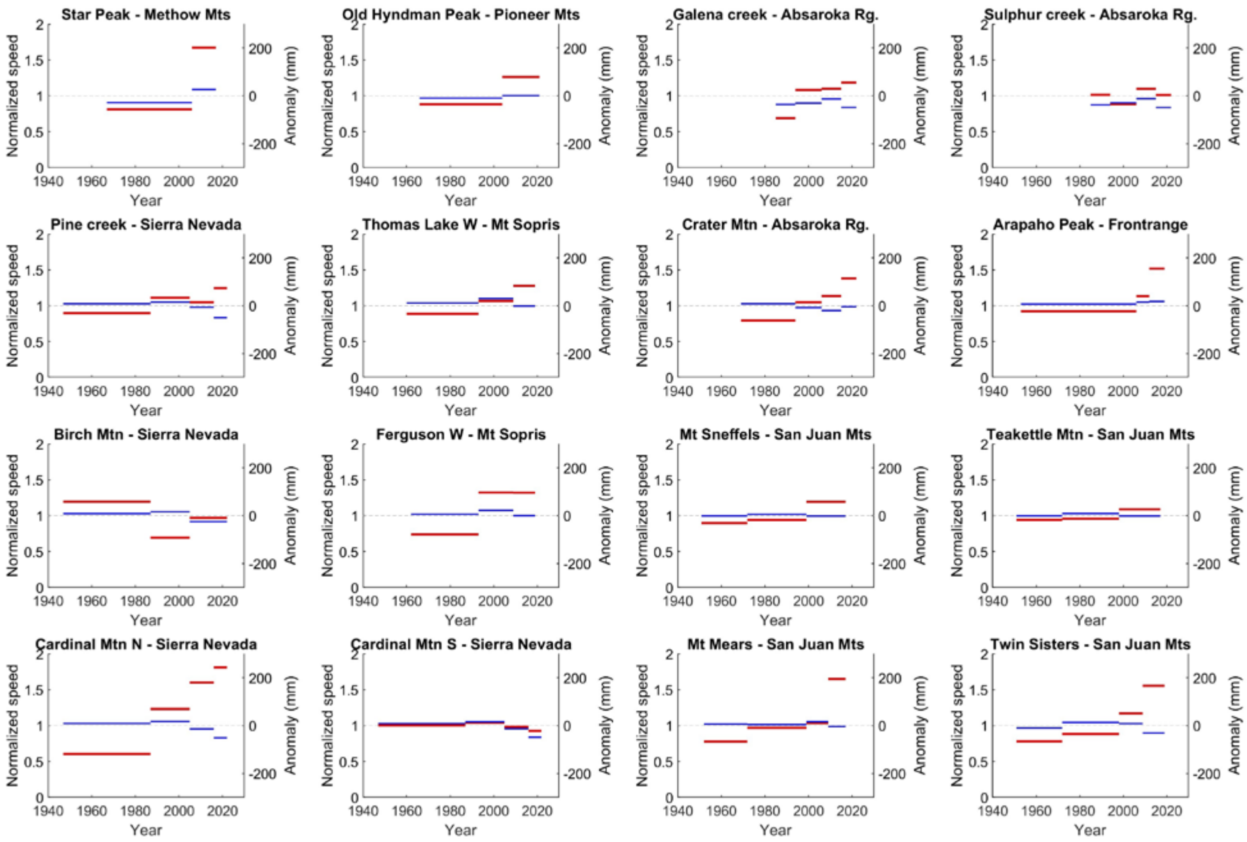

Figure S15: Rock glacier speed changes (red) and anomalies of total May-Oct precipitation for the intervals of the speed measurements (blue). Rock glacier speeds are the same as in Fig.2, air temperature anomaly means computed for different intervals than in Fig. S9.

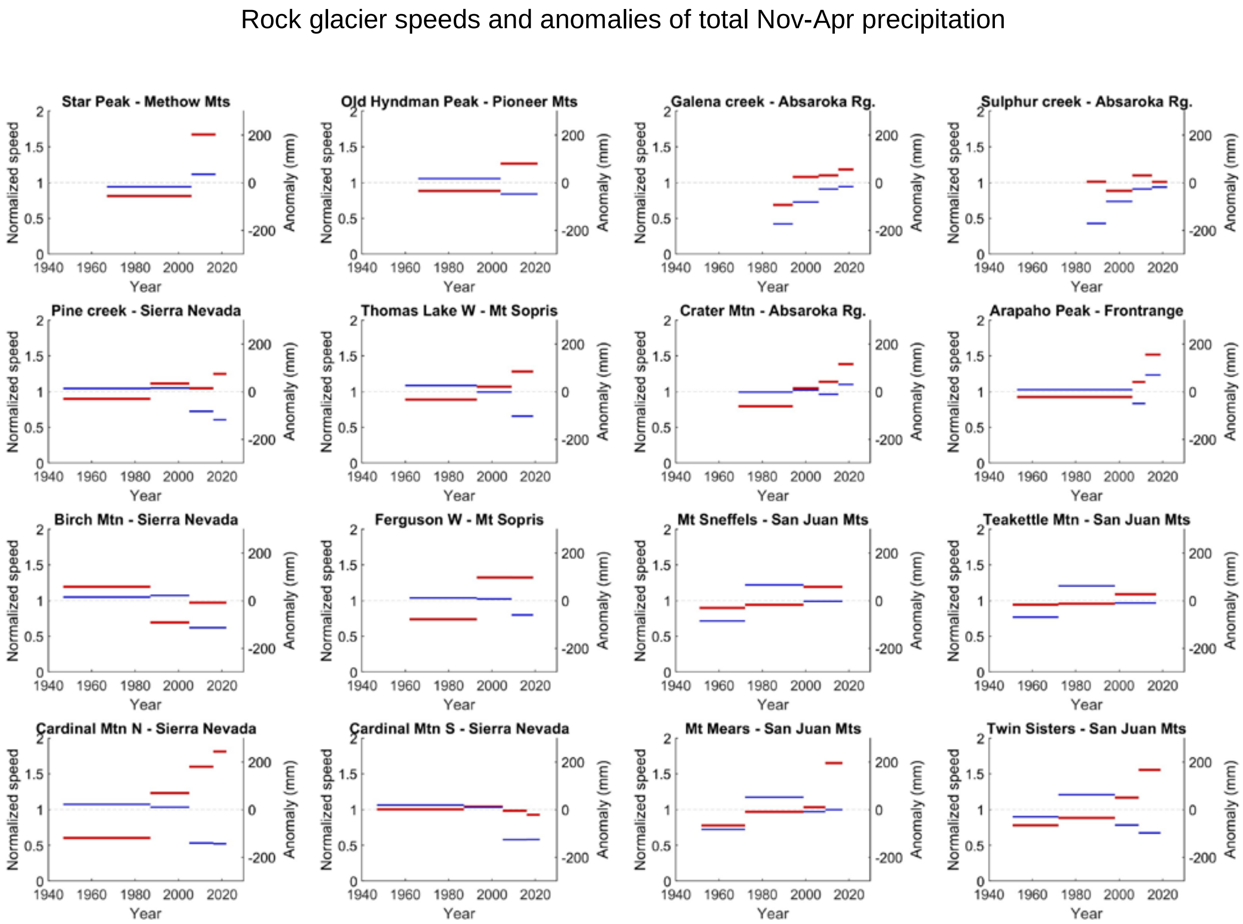

Figure S16: Rock glacier speed changes (red) and anomalies of total Nov-Oct precipitation for the intervals of the speed measurements (blue). Rock glacier speeds are the same as in Fig.2, air temperature anomaly means computed for different intervals than in Fig. S9.

Speed time series

Table S1: Median and normalized median speeds per time interval for all rock glaciers, and notches, as shown in Fig. 2. Mtn: Mountain, Mts: Mountains, Mt: Mount, N: North, S: South, W: West, Rg: Range.

**Star Peak - Methow Mts**					
Years	1967	2006	2017		
Med. speed (m/yr)	0,07	0,12			
Norm. med speed	0,81	1,67			
Notch	0,023	0,081			
**Old Hyndman Peak - Pioneer Mts**					
Years	1966	2004	2021		
Med. speed (m/yr)	0,21	0,28			
Norm. med speed	0,88	1,26			
Notch	0,033	0,073			
**Galena creek - Absaroka Rg.**					
Years	1985	1994	2006	2015	2022
Med. speed (m/yr)	0,33	0,53	0,55	0,61	
Norm. med speed	0,69	1,08	1,10	1,18	
Notch	0,090	0,099	0,080	0,068	
**Sulphur creek - Absaroka Rg.**					
Years	1985	1994	2006	2015	2022
Med. speed (m/yr)	0,42	0,38	0,49	0,33	
Norm. med speed	1,01	0,88	1,10	1,01	
Notch	0,105	0,049	0,089	0,106	
**Crater Mtn - Absaroka Rg.**					
Years	1969	1994	2006	2015	2022
Med. speed (m/yr)	0,35	0,46	0,50	0,57	
Norm. med speed	0,79	1,05	1,14	1,38	
Notch	0,013	0,028	0,02	0,031	
**Arapaho Peak - Frontrange**					
Years	1953	2006	2012	2019	
Med. speed (m/yr)	0,11	0,14	0,19		
Norm. med speed	0,92	1,13	1,52		
Notch	0,015	0,073	0,088		
**Thomas Lake W - Mt Sopris**					
Years	1960	1993	2009	2019	
Med. speed (m/yr)	0,56	0,62	0,83		
Norm. med speed	0,89	1,07	1,28		
Notch	0,011	0,03	0,05		
**Ferguson ranch W - Mt Sopris**					
Years	1962	1993	2009	2019	
Med. speed (m/yr)	0,52	0,89	0,97		
Norm. med speed	0,74	1,32	1,32		
Notch	0,077	0,107	0,088		
**Mt Mears - San Juan Mts**					
Years	1952	1972	1999	2009	2017
Med. speed (m/yr)	0,52	0,64	0,62	1,02	
Norm. med speed	0,78	0,97	1,03	1,65	
Notch	0,037	0,038	0,084	0,172	

Tab. S1 cont

Mt Sneffels - San Juan Mts					
Years	1951	1972	1999	2017	
Med. speed (m/yr)	0,43	0,42	0,59		
Norm. med speed	0,90	0,94	1,19		
Notch	0,025	0,014	0,029		
**Teakettle Mtn - San Juan Mts**					
Years	1951	1972	1998	2017	
Med. speed (m/yr)	0,21	0,20	0,23		
Norm. med speed	0,94	0,96	1,09		
Notch	0,017	0,013	0,015		
**Twin Sisters - San Juan Mts**					
Years	1951	1972	1998	2009	2019
Med. speed (m/yr)	0,37	0,43	0,59	0,77	
Norm. med speed	0,78	0,88	1,17	1,56	
Notch	0,056	0,026	0,033	0,094	
**Pine creek - Sierra Nevada**					
Years	1947	1987	2005	2016	2022
Med. speed (m/yr)	0,35	0,42	0,39	0,46	
Norm. med speed	0,90	1,11	1,05	1,25	
Notch	0,011	0,015	0,033	0,026	
**Birch Mtn - Sierra Nevada**					
Years	1947	1987	2005	2022	
Med. speed (m/yr)	0,44	0,24	0,35		
Norm. med speed	1,19	0,69	0,97		
Notch	0,035	0,045	0,080		
**Cardinal Mtn N - Sierra Nevada**					
Years	1947	1987	2005	2016	2022
Med. speed (m/yr)	0,35	0,74	0,85	0,82	
Norm. med speed	0,60	1,23	1,60	1,81	
Notch	0,027	0,017	0,038	0,082	
**Cardinal Mtn S - Sierra Nevada**					
Years	1947	1987	2005	2016	2022
Med. speed (m/yr)	0,61	0,61	0,59	0,53	
Norm. med speed	1,00	1,04	0,98	0,93	
Notch	0,015	0,017	0,036	0,039	

Information sheets for all rock glaciers

In the following, information sheets for all rock glaciers investigated are presented. The orthophoto maps are based on the U.S. Geological Survey / U.S. Department of Agriculture National Agricultural Imagery Program (USGS/USDA NAIP). White or black points indicate the measurement locations that were used to construct the speed time series. Topographic and climatic data are extracted from the rock glacier inventory by Johnson et al. (2021). Mean annual ground temperature is interpolated from the northern hemisphere permafrost model by Obu et al. (2019). The climate data in Johnsen et al. (2021) and the permafrost data by Obu et al. (2019) are both based on regional-scale data of 1 km spatial resolution and are thus *not* expected to precisely represent specific climatic and ground thermal conditions at individual rock glaciers. (For references see reference list in main text).

Star Peak – Methow Mts, WA

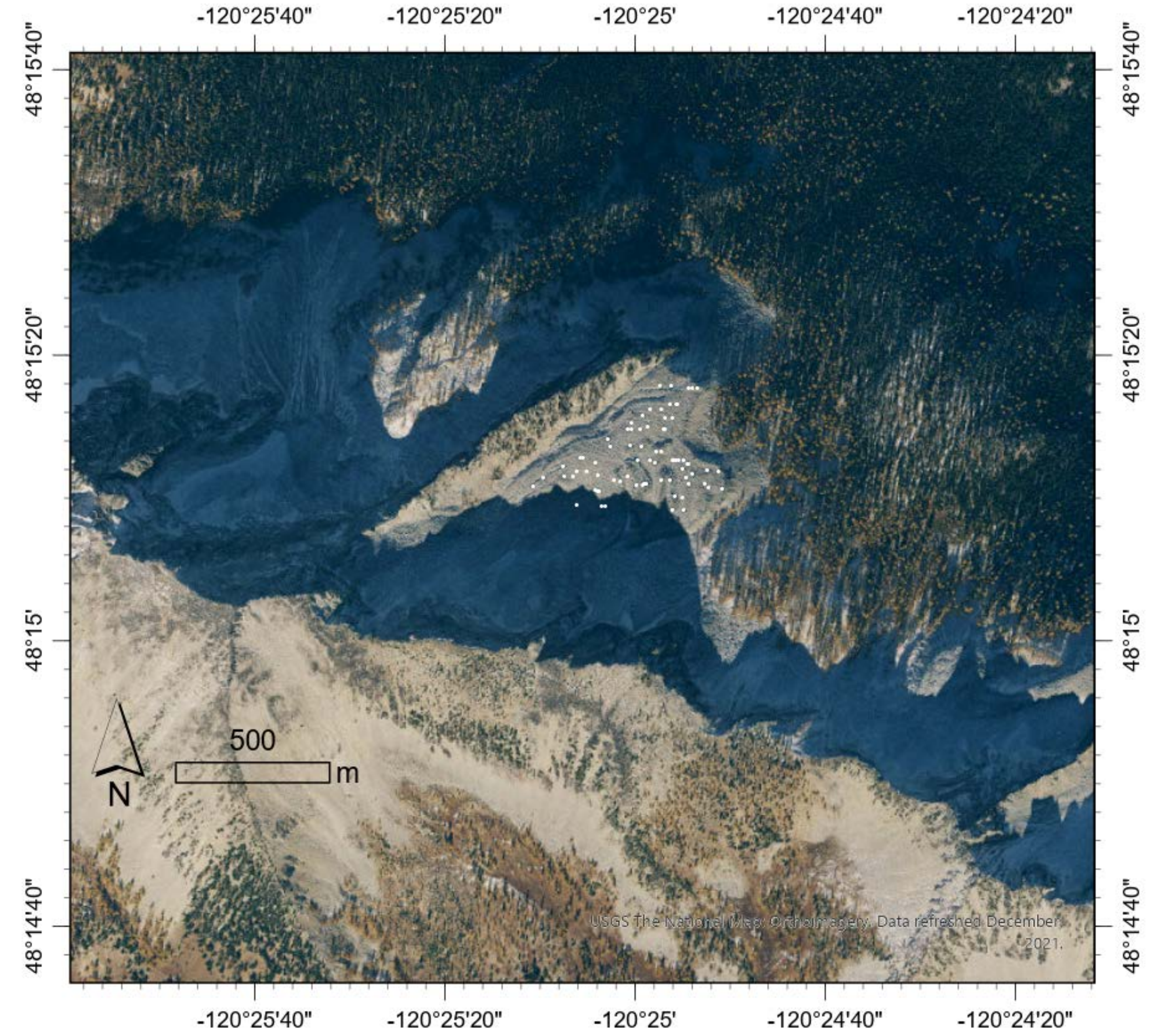

ELEV	SLOPE	PPT_WIN	PPT_SUM	PPT_ANN	SNO_ANN	TMEAN_WIN	TMEAN_SUM	TMEAN_ANN	MAGTM
2245	23	160	47	487	333	-6,8	9,4	0,8	2,4

Figure S17: Star Peak – Methow Mts, WA. White points indicate the measurement locations that are compiled to the speed time series. Airphoto courtesy of the U.S. Geological Survey (USDA NAIP). Data in the bottom row are from Johnson et al. (2021), from left to right: elevation (m), slope (deg), precipitation winter (mm, dec-feb), precipitation summer (mm, jun-aug), precipitation annual (mm), snow annual (mm SWE), mean temperature winter (deg C, dec-feb), mean temperature summer (deg C, jun-aug), mean temperature annual (deg C); from Obu et al. (2019): mean annual ground temperature (deg C).

Old Hyndman Peak – Pioneer Mts, ID

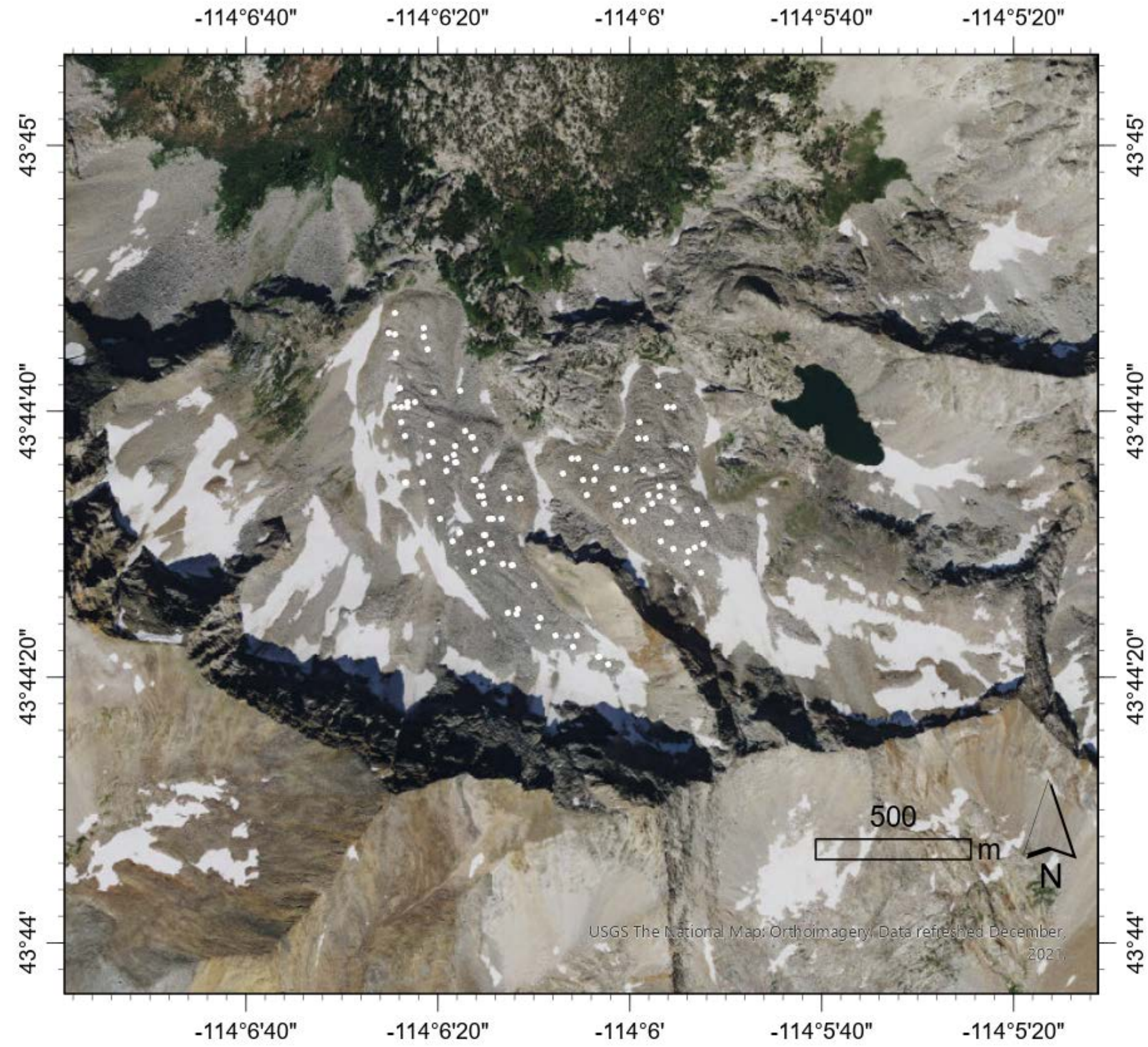

ELEV	SLOPE	PPT_WIN	PPT_SUM	PPT_ANN	SNO_ANN	TMEAN_WIN	TMEAN_SUM	TMEAN_ANN	MAGTM
3029	22	94	43	291	183	-8,9	9,4	-0,2	0,9

Figure S18: Old Hyndman Peak – Pioneer Mts, ID. White points indicate the measurement locations that are compiled to the speed time series. Points on both rock glaciers are included as they show similar behaviour. Airphoto courtesy of the U.S. Geological Survey (USDA NAIP). Data in the bottom row are from Johnson et al. (2021), from left to right: elevation (m), slope (deg), precipitation winter (mm, dec-feb), precipitation summer (mm, jun-aug), precipitation annual (mm), snow annual (mm SWE), mean temperature winter (deg C, dec-feb), mean temperature summer (deg C, jun-aug), mean temperature annual (deg C); from Obu et al. (2019): mean annual ground temperature (deg C).

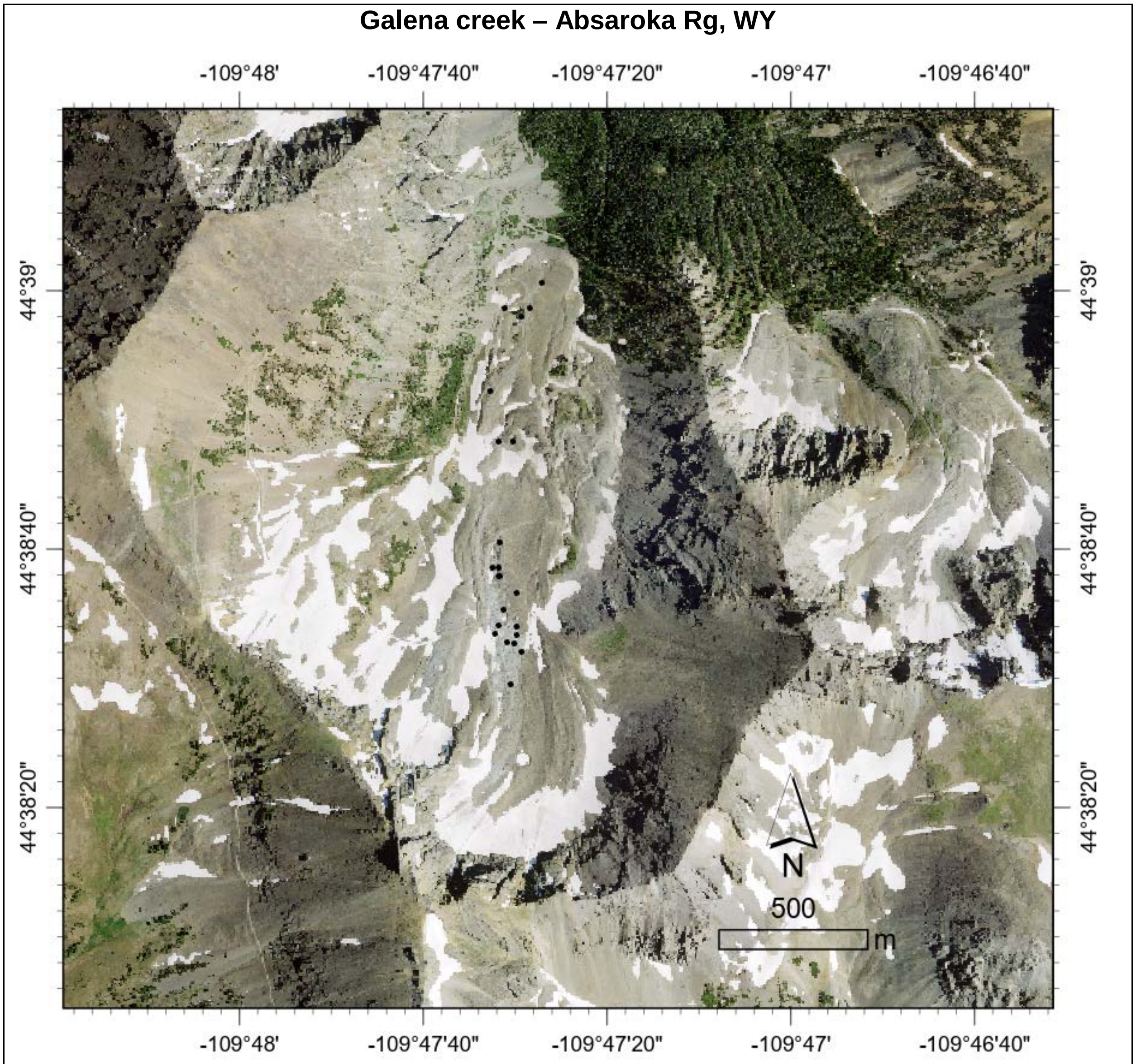

ELEV	SLOPE	PPT_WIN	PPT_SUM	PPT_ANN	SNO_ANN	TMEAN_WIN	TMEAN_SUM	TMEAN_ANN	MAGTM
2934	19	132	68	418	272	-9,7	9,4	-0,6	-0,5

Figure S19: Galena Creek – Absaroka Range, WY. Black points indicate the measurement locations that are compiled to the speed time series. Over large parts of the rock glacier surface debris is too fine to represent features that can be tracked in all airphotos. There is a rich body of research about Galena creek rock glacier (and also Sulphur rock glacier; see reference list of main text and references therein). Airphoto courtesy of the U.S. Geological Survey (USDA NAIP). Data in the bottom row are from Johnson et al. (2021), from left to right: elevation (m), slope (deg), precipitation winter (mm, dec-feb), precipitation summer (mm, jun-aug), precipitation annual (mm), snow annual (mm SWE), mean temperature winter (deg C, dec-feb), mean temperature summer (deg C, jun-aug), mean temperature annual (deg C); from Obu et al. (2019): mean annual ground temperature (deg C).

Sulphur creek – Absaroka Rg, WY

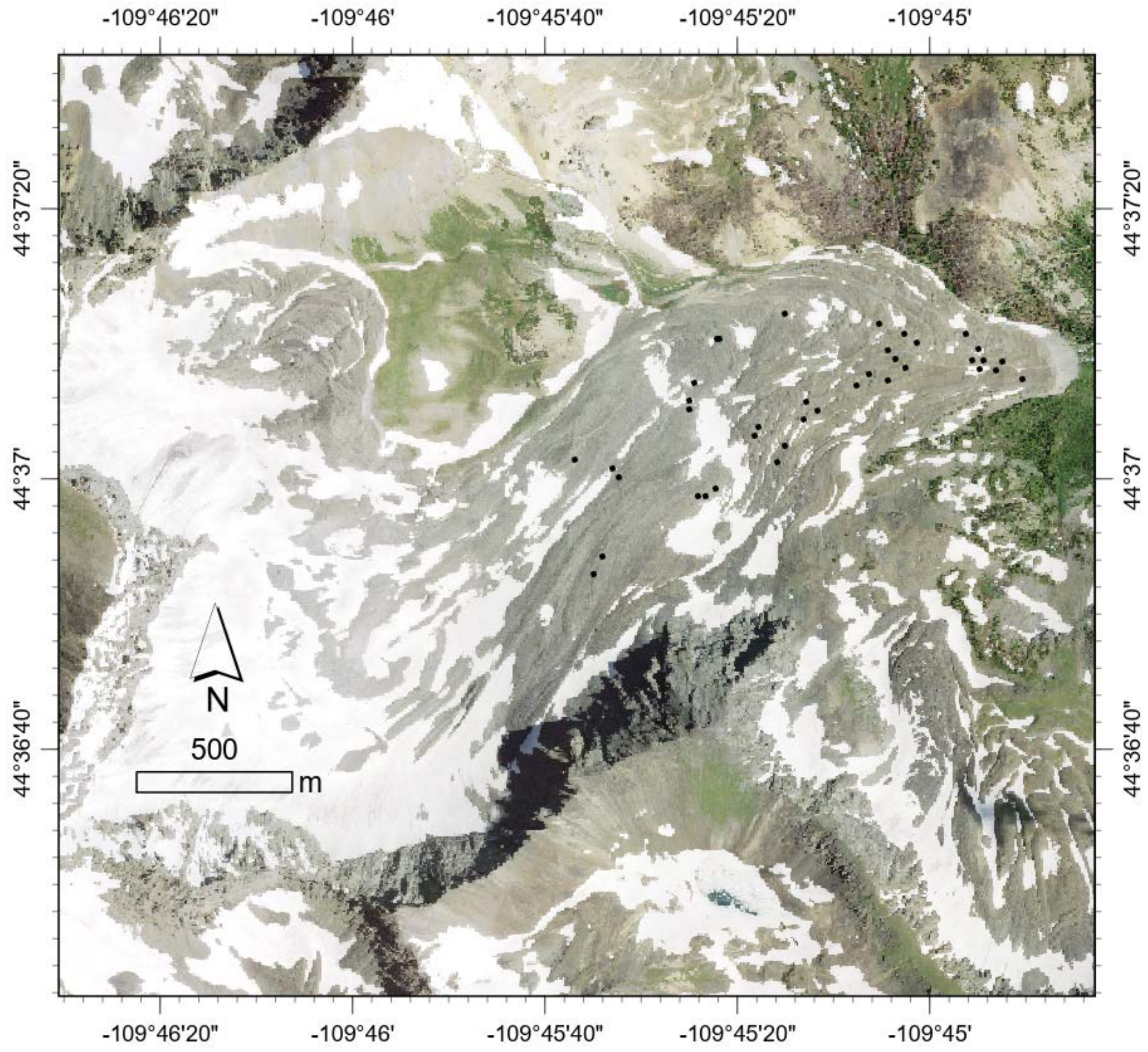

ELEV	SLOPE	PPT_WIN	PPT_SUM	PPT_ANN	SNO_ANN	TMEAN_WIN	TMEAN_SUM	TMEAN_ANN	MAGTM
3035	18	155	74	475	328	-10,1	8,8	-1,1	-1,0

Figure S20: Sulphur Creek – Absaroka Range, WY. Black points indicate the measurement locations that are compiled to the speed time series. Over large parts of the rock glacier surface debris is too fine to represent features that can be tracked in all airphotos. Airphoto courtesy of the U.S. Geological Survey (USDA NAIP). Data in the bottom row are from Johnson et al. (2021), from left to right: elevation (m), slope (deg), precipitation winter (mm, dec-feb), precipitation summer (mm, jun-aug), precipitation annual (mm), snow annual (mm SWE), mean temperature winter (deg C, dec-feb), mean temperature summer (deg C, jun-aug), mean temperature annual (deg C); from Obu et al. (2019): mean annual ground temperature (deg C).

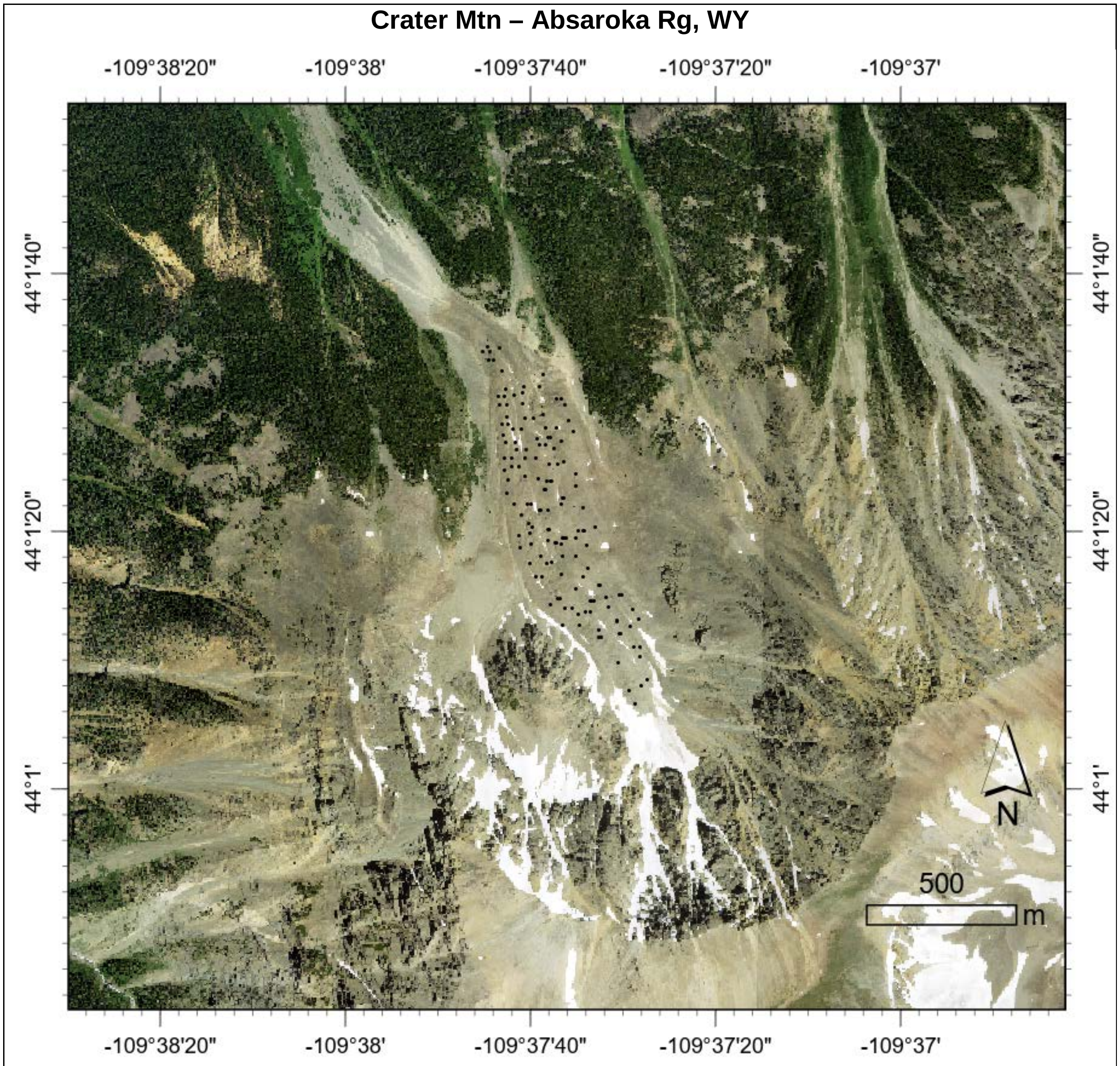

ELEV	SLOPE	PPT_WIN	PPT_SUM	PPT_ANN	SNO_ANN	TMEAN_WIN	TMEAN_SUM	TMEAN_ANN	MAGTM
2920	21	62	50	245	129	-8,7	10,5	0,3	1,0

Figure S21: Crater Mountain – Absaroka Range, WY. Black points indicate the measurement locations that are compiled to the speed time series. Airphoto courtesy of the U.S. Geological Survey (USDA NAIP). Data in the bottom row are from Johnson et al. (2021), from left to right: elevation (m), slope (deg), precipitation winter (mm, dec-feb), precipitation summer (mm, jun-aug), precipitation annual (mm), snow annual (mm SWE), mean temperature winter (deg C, dec-feb), mean temperature summer (deg C, jun-aug), mean temperature annual (deg C); from Obu et al. (2019): mean annual ground temperature (deg C).

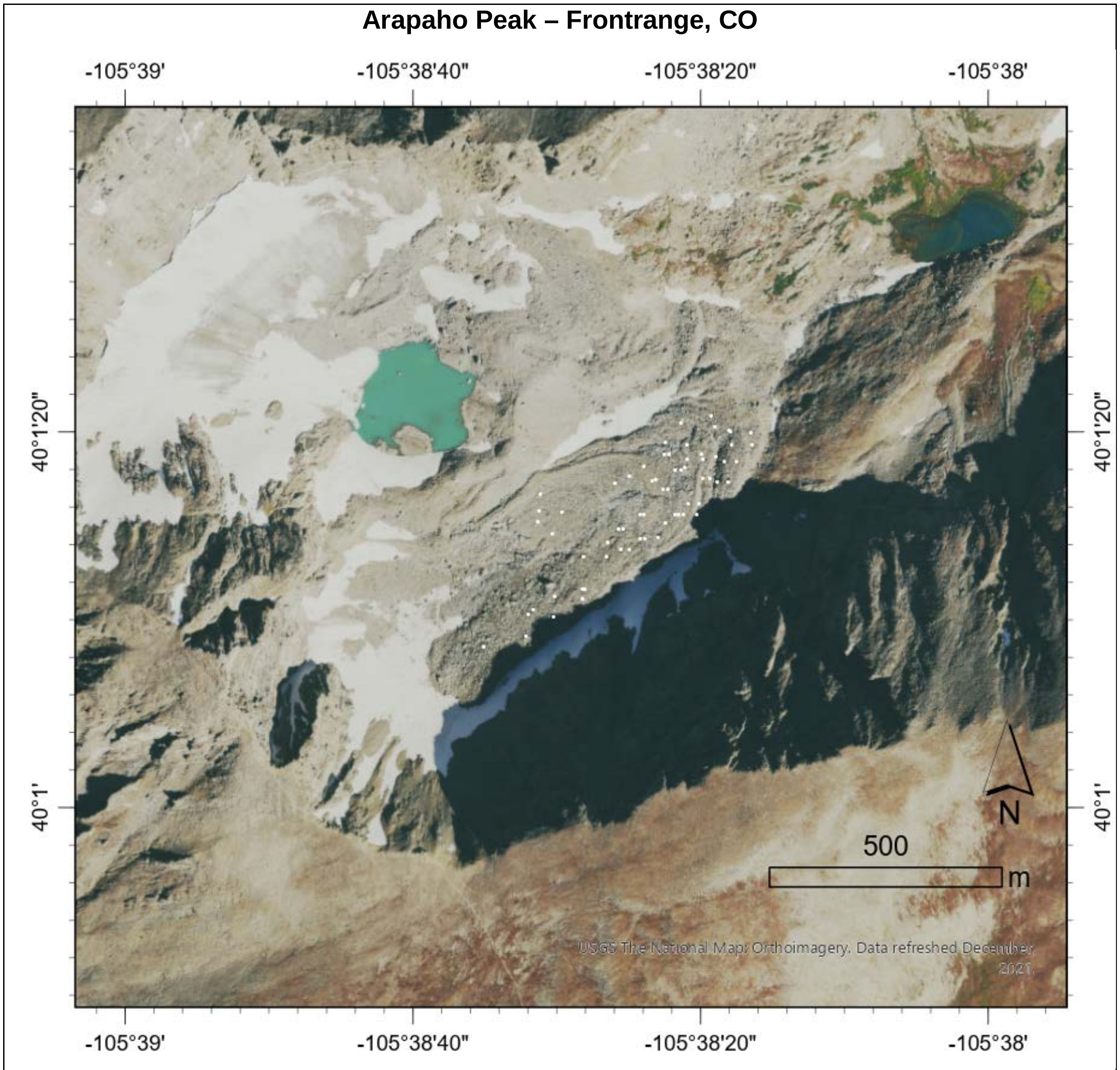

ELEV	SLOPE	PPT_WIN	PPT_SUM	PPT_ANN	SNO_ANN	TMEAN_WIN	TMEAN_SUM	TMEAN_ANN	MAGTM
3692	17	105	70	382	284	-11,1	6,6	-2,7	-2.7

Figure S22: Arapaho Peak – Frontrange, CO. White points indicate the measurement locations that are compiled to the speed time series. Airphoto courtesy of the U.S. Geological Survey (USDA NAIP). Data in the bottom row are from Johnson et al. (2021), from left to right: elevation (m), slope (deg), precipitation winter (mm, dec-feb), precipitation summer (mm, jun-aug), precipitation annual (mm), snow annual (mm SWE), mean temperature winter (deg C, dec-feb), mean temperature summer (deg C, jun-aug), mean temperature annual (deg C); from Obu et al. (2019): mean annual ground temperature (deg C).

Thomas lake W – Mt Sopris, CO

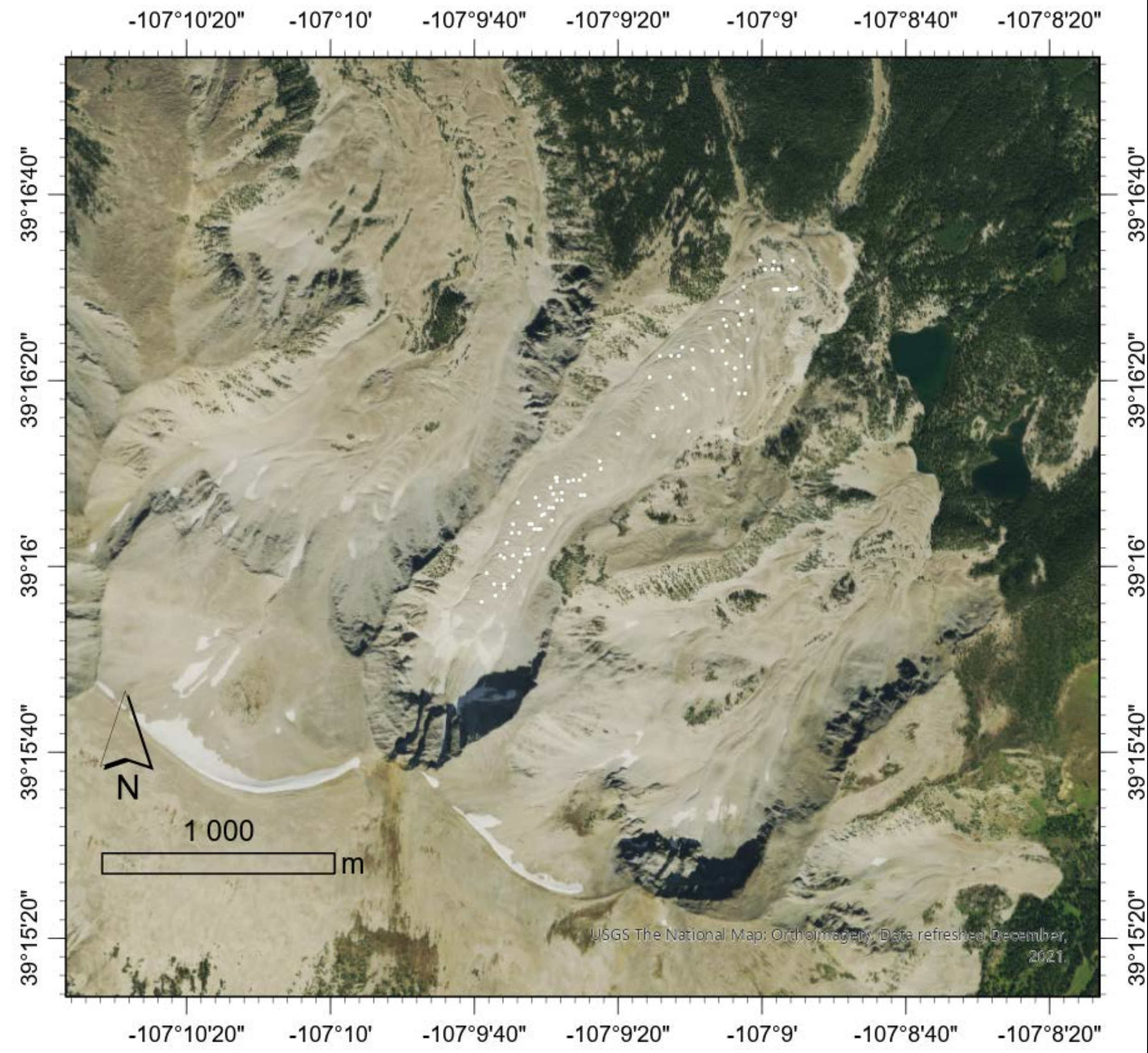

ELEV	SLOPE	PPT_WIN	PPT_SUM	PPT_ANN	SNO_ANN	TMEAN_WIN	TMEAN_SUM	TMEAN_ANN	MAGTM
3340	16	114	53	368	235	-9,1	12,2	0,8	1.4

Figure S23: Thomas lake West – Mount Sopris, CO. White points indicate the measurement locations that are compiled to the speed time series. Airphoto courtesy of the U.S. Geological Survey (USDA NAIP). Data in the bottom row are from Johnson et al. (2021), from left to right: elevation (m), slope (deg), precipitation winter (mm, dec-feb), precipitation summer (mm, jun-aug), precipitation annual (mm), snow annual (mm SWE), mean temperature winter (deg C, dec-feb), mean temperature summer (deg C, jun-aug), mean temperature annual (deg C); from Obu et al. (2019): mean annual ground temperature (deg C).

Ferguson ranch W – Mt Sopris, CO

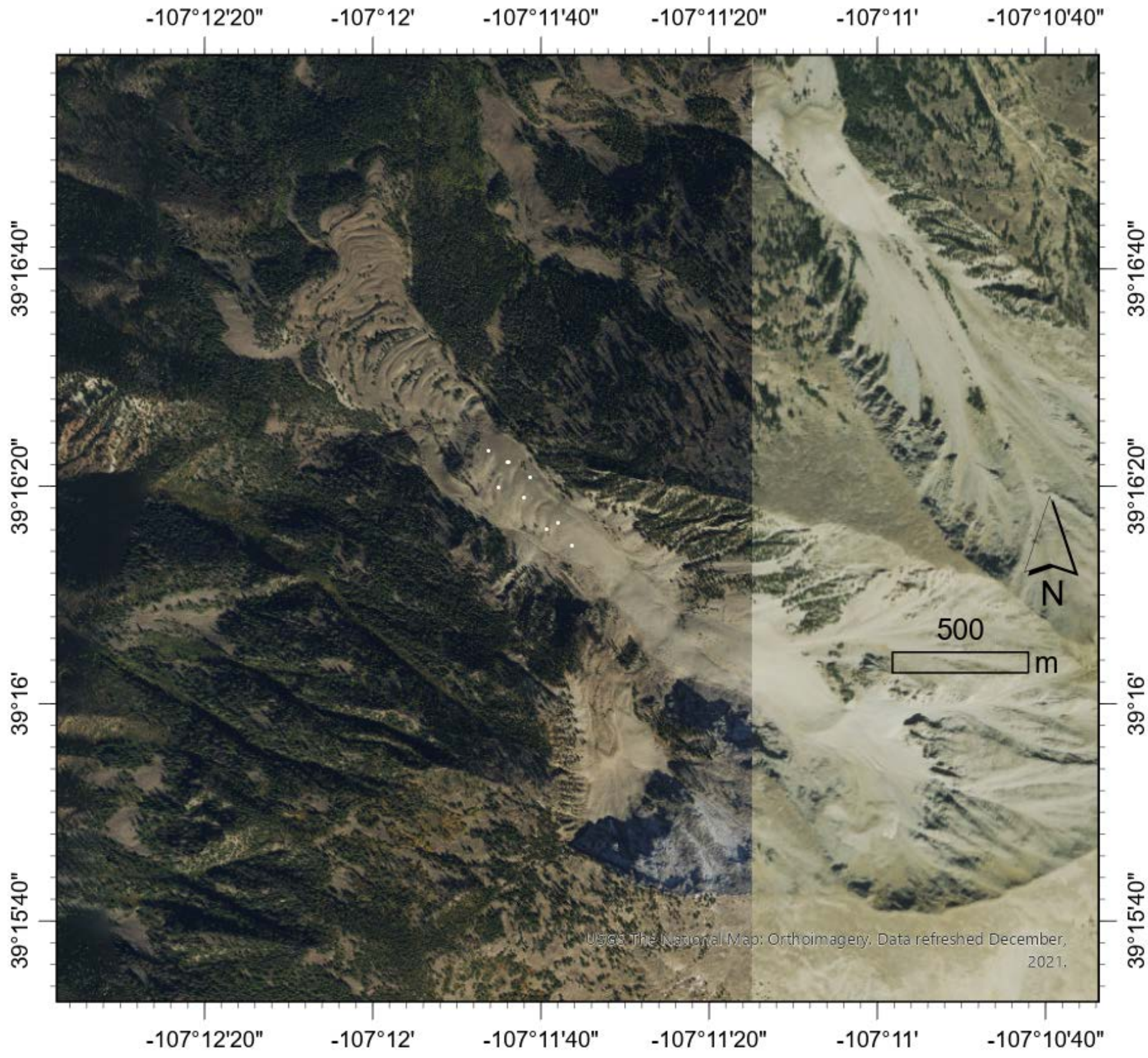

ELEV	SLOPE	PPT_WIN	PPT_SUM	PPT_ANN	SNO_ANN	TMEAN_WIN	TMEAN_SUM	TMEAN_ANN	MAGTM
2932	21	80	47	280	139	-6,7	14	3,3	4,3

Figure S24: Ferguson ranch West – Mount Sopris, CO. White points indicate the measurement locations that are compiled to the speed time series. Over large parts of the rock glacier surface debris is too fine to represent features that can be tracked in all airphotos. Airphoto courtesy of the U.S. Geological Survey (USDA NAIP). Data in the bottom row are from Johnson et al. (2021), from left to right: elevation (m), slope (deg), precipitation winter (mm, dec-feb), precipitation summer (mm, jun-aug), precipitation annual (mm), snow annual (mm SWE), mean temperature winter (deg C, dec-feb), mean temperature summer (deg C, jun-aug), mean temperature annual (deg C); from Obu et al. (2019): mean annual ground temperature (deg C).

Mt Mears – San Juan Mts, CO

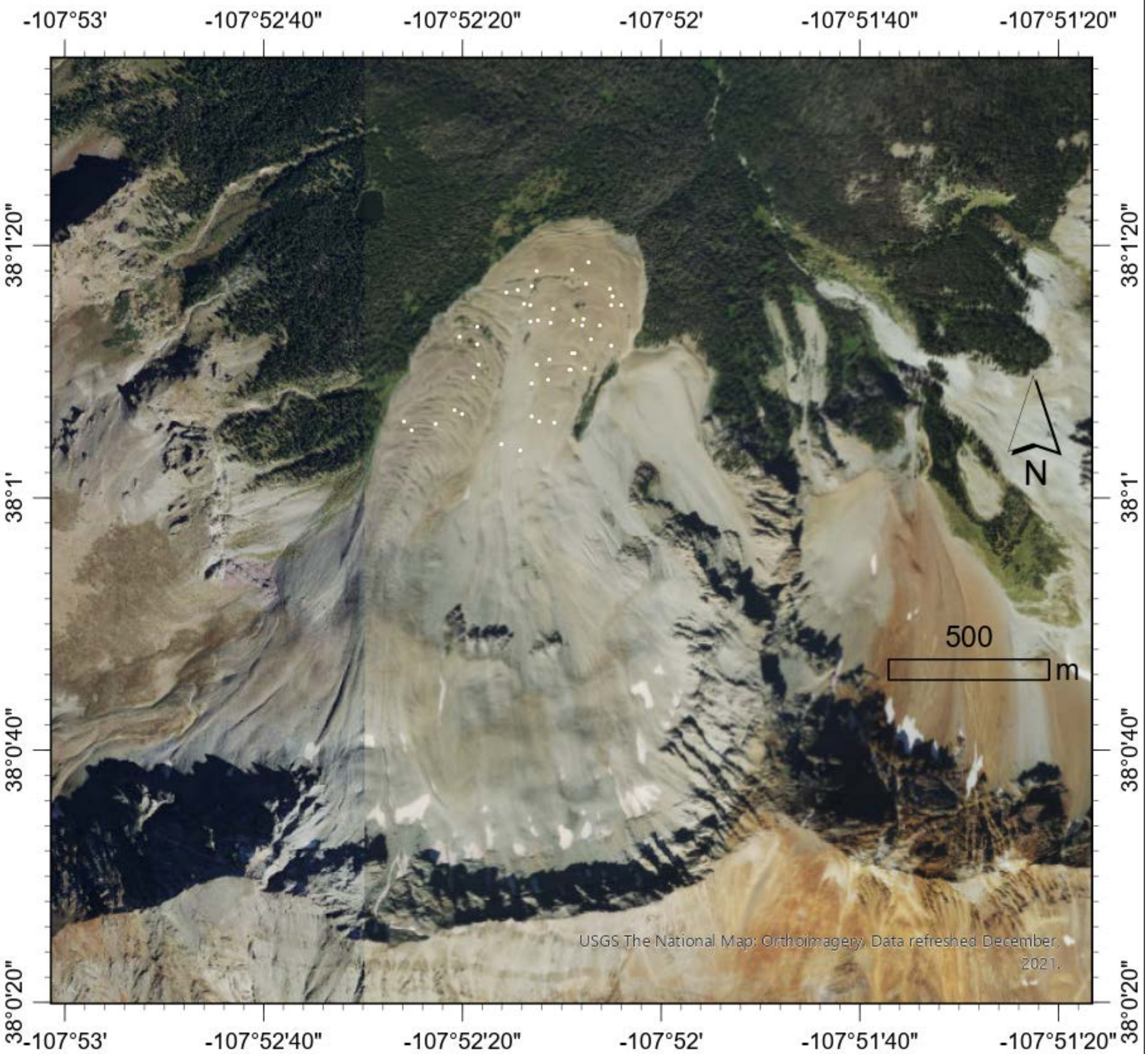

ELEV	SLOPE	PPT_WIN	PPT_SUM	PPT_ANN	SNO_ANN	TMEAN_WIN	TMEAN_SUM	TMEAN_ANN	MAGTM
3447	21	98	72	347	192	-6,8	10,4	1,4	1,1

Figure S25: Mount Mears – San Juan Mountains, CO. White points indicate the measurement locations that are compiled to the speed time series. Over large parts of the rock glacier surface debris is too fine to represent features that can be tracked in all airphotos. Airphoto courtesy of the U.S. Geological Survey (USDA NAIP). Data in the bottom row are from Johnson et al. (2021), from left to right: elevation (m), slope (deg), precipitation winter (mm, dec-feb), precipitation summer (mm, jun-aug), precipitation annual (mm), snow annual (mm SWE), mean temperature winter (deg C, dec-feb), mean temperature summer (deg C, jun-aug), mean temperature annual (deg C); from Obu et al. (2019): mean annual ground temperature (deg C).

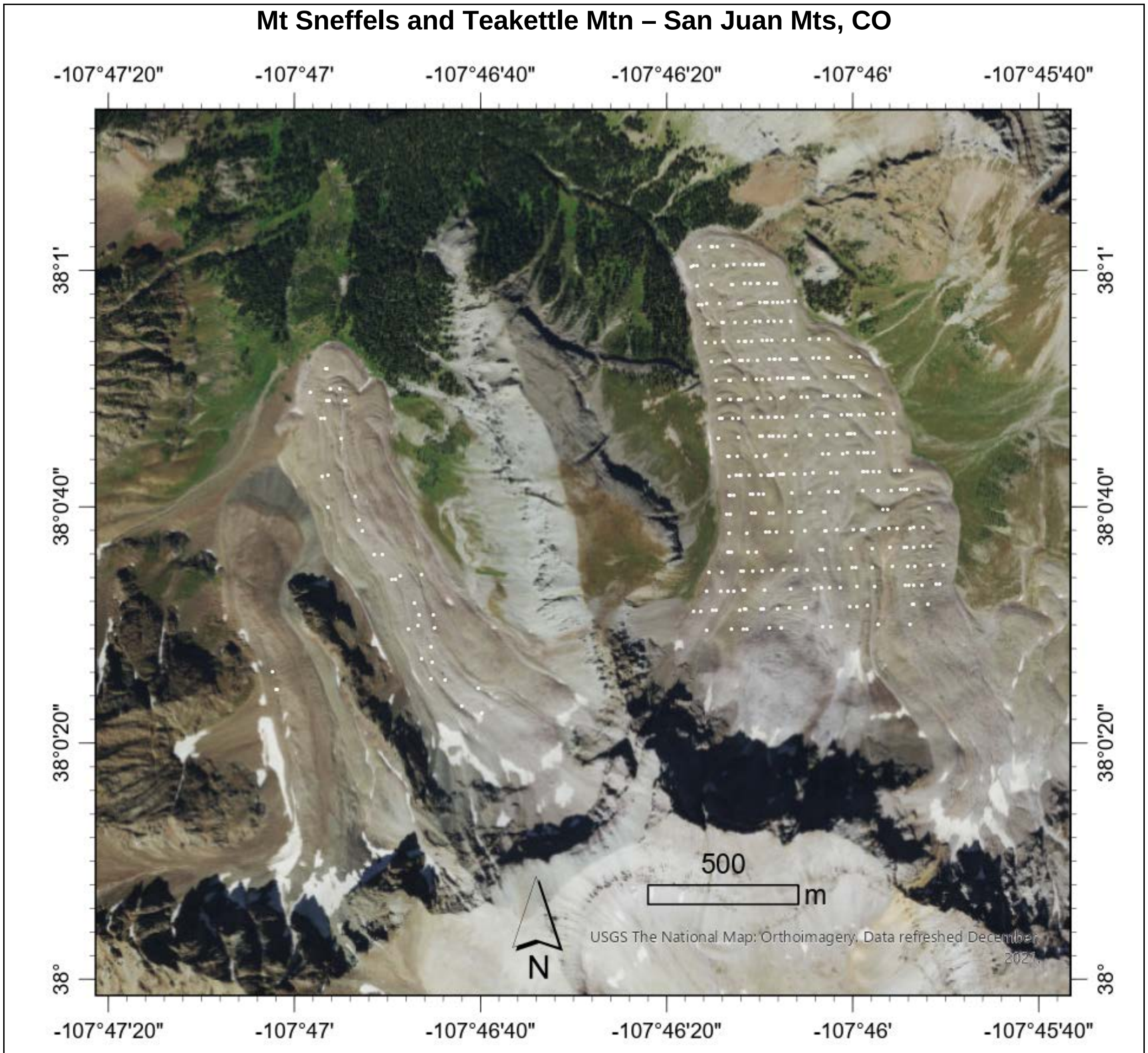

ELEV	SLOPE	PPT_WIN	PPT_SUM	PPT_ANN	SNO_ANN	TMEAN_WIN	TMEAN_SUM	TMEAN_ANN	MAGTM
3587	20	109	72	369	217	-7,7	9,3	0,4	0,2
3653	16	110	73	373	220	-8,4	8,6	-0,3	-0,3

Figure S26: Mount Sneffels and Teakettle Mountain – San Juan Mountains, CO. Sneffels rock glacier left, Teakettle rock glacier right. White points indicate the measurement locations that are compiled to the speed time series. Over large parts of the Sneffels rock glacier surface debris is too fine to represent features that can be tracked in all airphotos. Teakettle Mtn rock glacier was the only rock glacier of the study where raster-based measurement locations where partially successful. Airphoto courtesy of the U.S. Geological Survey (USDA NAIP). Data in the bottom row are from Johnson et al. (2021), from left to right: elevation (m), slope (deg), precipitation winter (mm, dec-feb), precipitation summer (mm, jun-aug), precipitation annual (mm), snow annual (mm SWE), mean temperature winter (deg C, dec-feb), mean temperature summer (deg C, jun-aug), mean temperature annual (deg C); from Obu et al. (2019): mean annual ground temperature (deg C). Sneffels rock glacier upper line, Teakettle rock glacier lower line.

Twin Sisters – San Juan Mts, CO

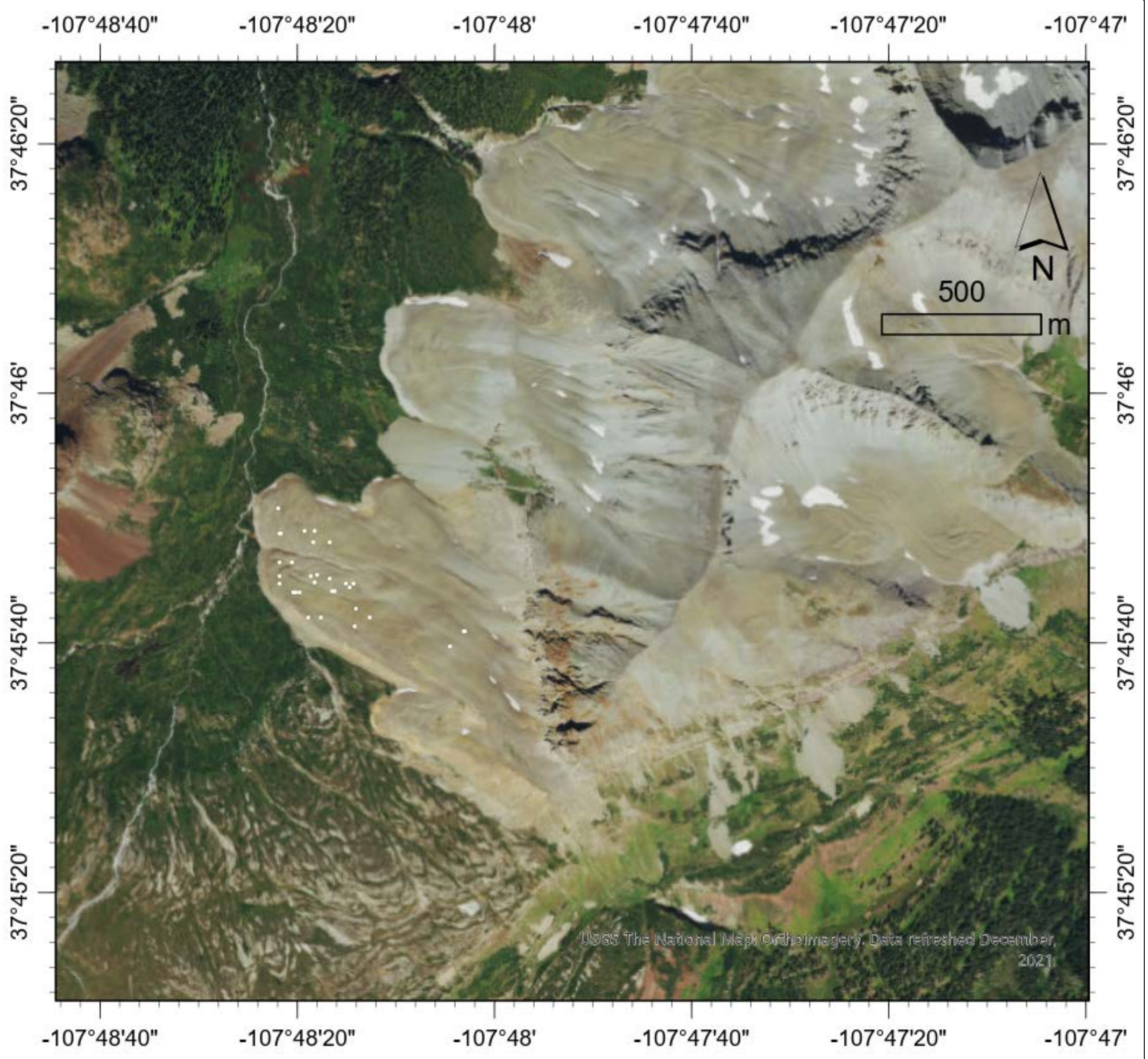

ELEV	SLOPE	PPT_WIN	PPT_SUM	PPT_ANN	SNO_ANN	TMEAN_WIN	TMEAN_SUM	TMEAN_ANN	MAGTM
3601	23	131	68	414	256	-7,7	9,1	0,3	-0,2

Figure S27: Twin Sisters – San Juan Mountains, CO. Twin Sisters rock glacier to the lower left. White points indicate the measurement locations that are compiled to the speed time series. Over large parts of the rock glacier surface debris is too fine to represent features that can be tracked in all airphotos. Airphoto courtesy of the U.S. Geological Survey (USDA NAIP). Data in the bottom row are from Johnson et al. (2021), from left to right: elevation (m), slope (deg), precipitation winter (mm, dec-feb), precipitation summer (mm, jun-aug), precipitation annual (mm), snow annual (mm SWE), mean temperature winter (deg C, dec-feb), mean temperature summer (deg C, jun-aug), mean temperature annual (deg C); from Obu et al. (2019): mean annual ground temperature (deg C).

Pine creek and Birch Mtn – Sierra Nevada, CA

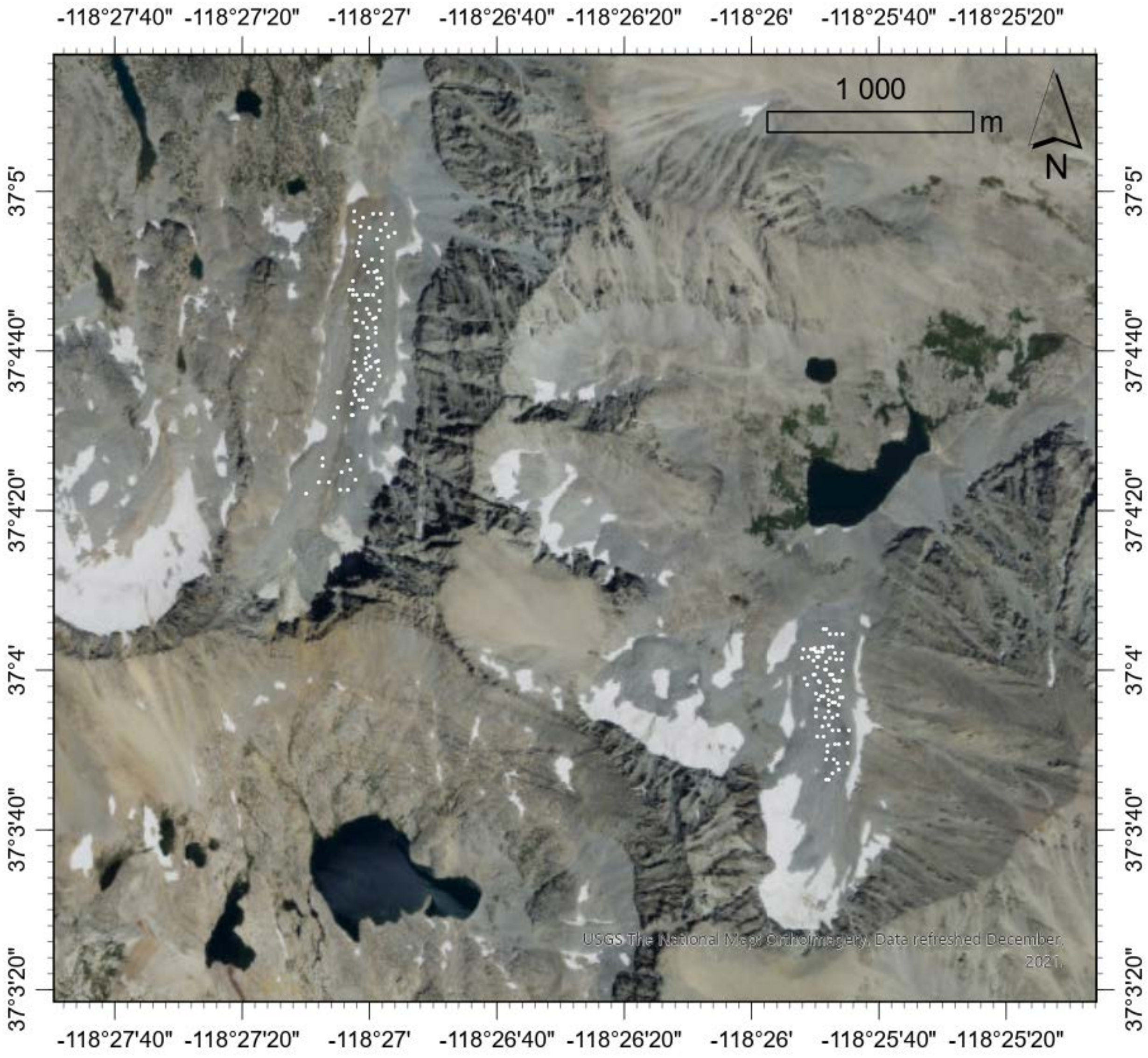

ELEV	SLOPE	PPT_WIN	PPT_SUM	PPT_ANN	SNO_ANN	TMEAN_WIN	TMEAN_SUM	TMEAN_ANN	MAGTM
3516	13	182	16	360	310	-6,7	7,3	-0,4	0,3
3595	17	184	17	367	319	-7,2	7,2	-0,7	0,9

Figure S28: Pine creek and Birch Mountain – Sierra Nevada, CA. Pine creek is left, Birch Mtn is right rock glacier. White points indicate the measurement locations that are compiled to the speed time series. Airphoto courtesy of the U.S. Geological Survey (USDA NAIP). Data in the bottom row are from Johnson et al. (2021), from left to right: elevation (m), slope (deg), precipitation winter (mm, dec-feb), precipitation summer (mm, jun-aug), precipitation annual (mm), snow annual (mm SWE), mean temperature winter (deg C, dec-feb), mean temperature summer (deg C, jun-aug), mean temperature annual (deg C); from Obu et al. (2019): mean annual ground temperature (deg C). Pine creek upper line, Birch Mtn lower line.

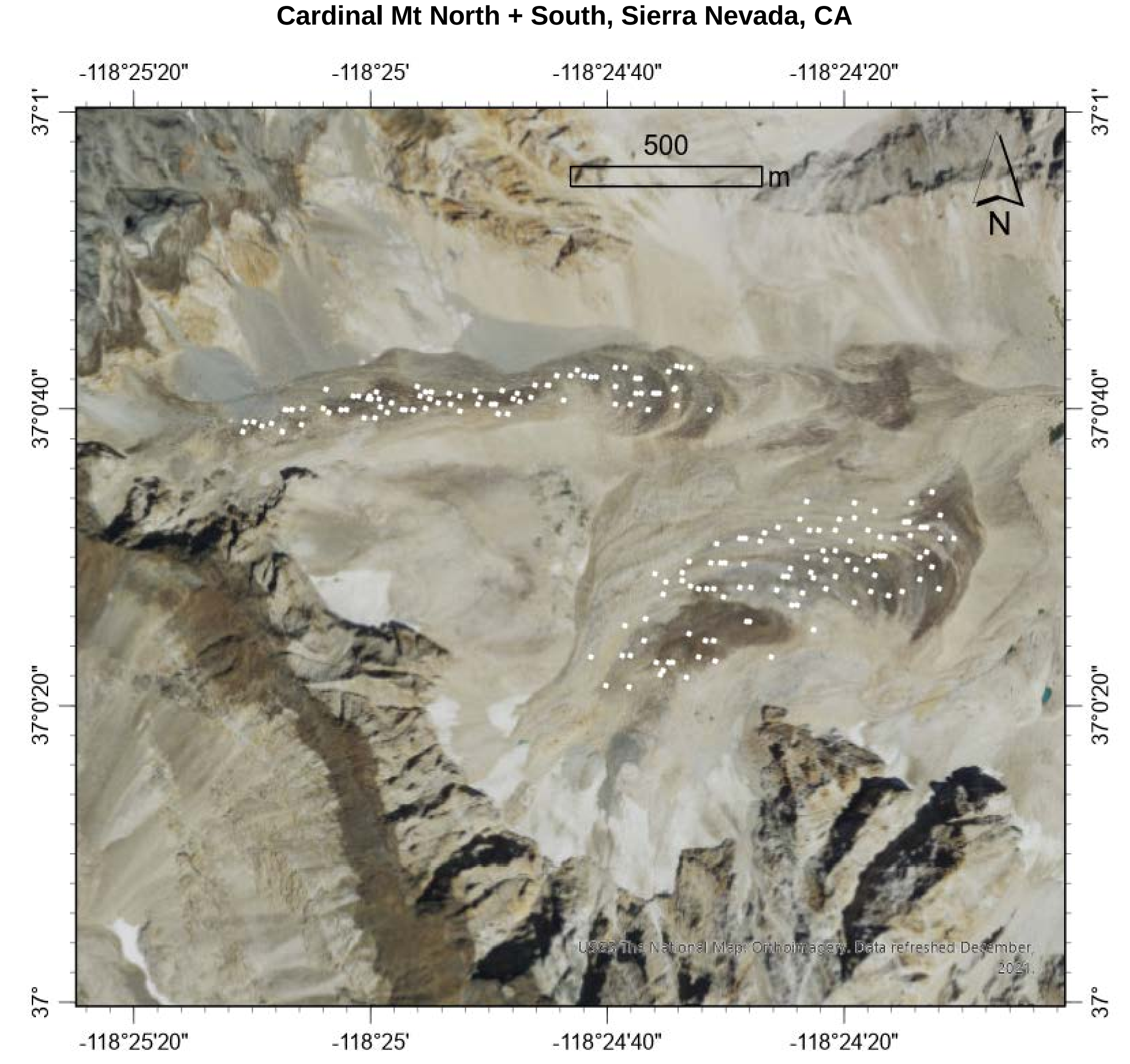

ELEV	SLOPE	PPT_WIN	PPT_SUM	PPT_ANN	SNO_ANN	TMEAN_WIN	TMEAN_SUM	TMEAN_ANN	MAGTM
2920	21	62	50	245	129	-8,7	10,5	0,3	0,9

Figure S29: Cardinal Mountain North + South – Sierra Nevada, CA. Cardinal North is upper, Cardinal South is lower rock glacier. White points indicate the measurement locations that are averaged to the speed time series. Airphoto courtesy of the U.S. Geological Survey (USDA NAIP). Data in the bottom row are from Johnson et al. (2021), from left to right: elevation (m), slope (deg), precipitation winter (mm, dec-feb), precipitation summer (mm, jun-aug), precipitation annual (mm), snow annual (mm SWE), mean temperature winter (deg C, dec-feb), mean temperature summer (deg C, jun-aug), mean temperature annual (deg C); from Obu et al. (2019): mean annual ground temperature (deg C). Values apply to both rock glaciers.

List of airphotos

Table S2: Years of photography and USGS identifiers of all airphotos used from the USGS Earth Explorer data base. Airphotos are from the following programmes and collections: National Agricultural Imagery Program (NAIP); National Aerial Photography Program (NAPP) and Aerial Photo Single Frames. For Arapaho and Star Peak also special acquisitions from the High Resolution Orthoimagery collection are available. See USGS Earth Explorer documentation for information about these programmes and collections.

	Year	Image ID
Star Peak - Methow Mts		
	1967	1VBTU00010230
	2006	n_4812045_se_10_1_20060701
	2017	m_4812045_se_10_1_20171015
Old Hyndman Peak - Pioneer Mts		
	1966	1VBNS00020225
	2004	n_4311424_nw_11_1_20040807
	2021	m_4311424_nw_11_060_20210915
Galena and Sulphur creek - Absaroka Rg.		
	1985	1VFGJC0040035
	1994	O4410926.NES.831145; O4410918.SES.831159; n_4410918_se_12_1_20060902;
	2006	n_4410926_ne_12_1_20060902 m_4410926_ne_12_h_20150924;
	2015	m_4410918_se_12_h_20150924 m_4410918_se_12_060_20220716;
	2022	m_4410926_ne_12_060_20220716
Crater Mtn - Absaroka Rg.		
	1969	1VCFV00010063
	1994	O4410959.SES.833213
	2006	n_4410959_se_12_1_20060902
	2015	m_4410959_se_12_h_20150923
	2022	m_4410959_se_12_060_20220710
Arapaho Peak - Frontrange		
	1953	1VAP000030162
	2006	O4010559.SES.1147654
	2012	001n074w026
	2019	m_4010559_se_13_060_20190926
Ferguson ranch W and Thomas Lake W - Mt Sopris		
	1962/60	1VAMZ00010125; 1VAAM00020122
	1993	O3910747.SWS.1023248; O3910747.SES.1023247
	2009	m_3910747_sw_13_1_20090717; m_3910747_se_13_1_20090812
	2019	m_3910747_sw_13_060_20190914; m_3910747_se_13_060_20190914;
Mt Mears - San Juan Mts		
	1952	1VC0000010075
	1972	6213005900050
	1999	N10NAPPW11654130
	2009	m_3810758_sw_13_1_20090727
	2017	m_3810758_sw_13_1_20170921
Mt Sneffels and Teakettle Mtn - San Juan Mts		
	1951	1PR0000010124
	1972	6213005900097
	1999	N10NAPPW11392069
	2009	m_3810758_se_13_1_20090727
	2017	m_3810758_se_13_1_20170925

Tab S2 cont.

Twin Sisters - San Juan Mts		
	1951	1PR0000010005
	1972	6213005900069
	1998	1998_O3710710.SES.1022243
	2009	m_3710710_se_13_1_20090727
	2019	m_3710710_se_13_060_20190909
Pine creek and Birch Mtn- Sierra Nevada		
	1947	1CQ0000060149; 1CQ0000060150; 1CQ0000060151; 1CQ0000060152
	1987	C3711861.NWS.865921; C3711861.NES.865917
	2005	n_3711861_nw_11_1_2005082; n_3711861_ne_11_1_20050828
	2016	m_3711861_nw_11_h_20160726; m_3711861_ne_11_h_20160726
	2022	m_3711861_nw_11_060_20220709; m_3711861_ne_11_060_20220709
Cardinal Mtn N and S - Sierra Nevada		
	1947	1CQ0000060149; 1CQ0000060150; 1CQ0000060151; 1CQ0000060152;
	1987	C3711861.SES.865926
	2005	n_3711861_se_11_1_20050828
	2016	m_3711861_se_11_h_20160726
	2022	m_3711861_se_11_060_20220709
